# A Hybrid Response Surface Methodology and Machine Learning Framework for Quantifying Effects of Physicochemical Parameters on PFAS Distribution

**DOI:** 10.1021/acsestwater.5c01162

**Published:** 2026-04-17

**Authors:** Harsh V. Patel, Jazmin Green, Hyoshin Park, Stephanie Luster-Teasley Pass, Renzun Zhao

**Affiliations:** † Department of Civil, Architectural, and Environmental Engineering, 3616 North Carolina A&T State University, Greensboro, North Carolina 27411, United States; ‡ Department of Computer Science, North Carolina A&T State University, Greensboro, North Carolina 27411, United States; § Department of Engineering Management and Systems Engineering, 6042 Old Dominion University, Norfolk, Virginia 23529, United States

**Keywords:** per- and polyfluoroalkyl substance (PFAS), machine learning, response surface methodology, adsorption, distribution coefficient

## Abstract

Predicting PFAS adsorption across diverse adsorbents and environmental matrices remains challenging because adsorbent physicochemical properties, PFAS molecular descriptors, and operational conditions simultaneously influence adsorption. This study develops and evaluates a unified hybrid modeling framework that integrates Response Surface Model (RSM) with machine-learning algorithms to quantify how six key variables, surface area, Log *K*_ow_, pH_pzc_, p*K*_a_, log dose, and log-initial concentration, affect PFAS distribution coefficients (Log *K*_d_). A data set of more than 1000 adsorption observations spanning 15 PFAS compounds, multiple adsorbent types, and a broad operational range was compiled and preprocessed using mode imputation and log transformation. Model performance was evaluated using an 80/20 split and Leave-One-PFAS-Out (LOPO) validation. Gradient Boosting performed best in the 80/20 scenario (*R* ^2^ = 0.93; RMSE = 0.25), whereas Random Forest achieved the highest performance under LOPO validation (*R* ^2^ = 0.30; RMSE = 0.78), highlighting the challenge of compound-wise generalization. Feature-importance analyses consistently identify log-dose and log-initial concentration as key predictors, followed by surface area and pHpzc. Partial-dependence analysis and RSM surfaces revealed nonlinear relationships and interaction effects among adsorption drivers. Overall, the framework provides a transparent approach for predicting PFAS adsorption while disentangling the coupled roles of adsorbent properties, PFAS chemistry, and operational conditions.

## Graphical abstract

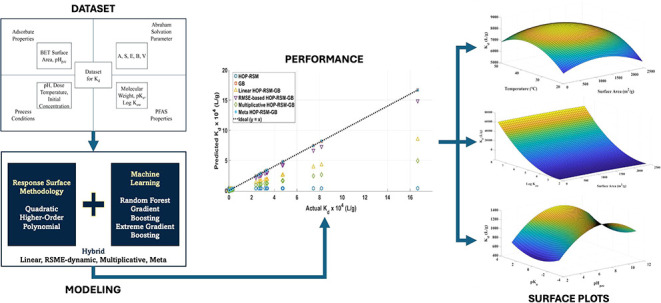

## 1 Introduction

Per- and polyfluoroalkyl substances (PFAS) have emerged as critical contaminants due to their extreme environmental persistence, aqueous mobility, and well-documented toxicological concerns.^1–5^ As of May 14th, 2025, the USA EPA will maintain the National Drinking Water Regulations for perfluorooctanoic acid (PFOA) and perfluorooctanesulfonic acid (PFOS) set at 4 ng/L each.^6^ These limits underscore the urgency of developing effective remediation strategies to protect public health and comply with regulatory standards. Their strong C–F bonds and amphiphilic molecular structures make PFAS highly resistant to conventional treatment processes, placing increasing pressure on engineered systems to deliver reliable removal from drinking water, wastewater, and landfill leachate. Adsorption remains one of the most widely implemented treatment strategies; however, PFAS adsorption behavior varies substantially across adsorbent classes, PFAS chemistries, and operational conditions, complicating efforts to predict treatment performance and optimize design (McNamara et al., 2018; Pramanik et al., 2015; Ross et al., 2018).

This variability is amplified by the fragmented nature of the existing literature. Most adsorption studies investigate single PFAS–adsorbent combinations under narrow ranges of pH, dose, concentration, and ionic strength, producing data sets that are difficult to generalize or compare (Ross et al., 2018). Traditional modeling approaches—including isotherm fitting and response surface methodology (RSM)—provide mechanistic transparency but struggle to capture the nonlinear, multidimensional interactions that govern PFAS adsorption in refs. 7–12 In PFAS studies, RSM has been used to optimize degradation and adsorption processes, including PFOS removal via heterogeneous photocatalysis (83% removal in 8 h),^13^ PFOA adsorption using alginate hydrogel (94.8 ± 2.1% removal),^14^ and PFOA removal with layered double hydroxides and peroxydisulfate activation.^15^ Meanwhile, machine learning (ML) offers strong predictive capabilities but often lacks interpretability and can perform poorly when extrapolating to PFAS not represented in the training data set (Karbassiyazdi et al.^16^).

Here, we integrate quadratic RSM with advanced ML algorithms to develop an interpretable, data-driven framework for predicting PFAS distribution coefficients (log *K*_d_) across diverse PFAS classes, adsorbent types, and operational conditions. A consolidated data set (>1000 observations, 15 PFAS) was preprocessed using mode imputation and log-transformed operational variables to represent key physicochemical and treatment-relevant predictors. Model robustness was evaluated using both a conventional 80/20 split and a stringent leave-one-PFAS-out (LOPO) validation designed to directly test chemical transferability.

Accordingly, this study aims to develop a unified and interpretable modeling framework for predicting PFAS adsorption across diverse chemistries and adsorbent materials. The specific objectives are to:

- Quantify the individual and interactive effects of adsorbent properties, PFAS molecular characteristics, and operational conditions on the distribution coefficient (log *K*_d_) using Response Surface Model (RSM).
- Develop and benchmark advanced machine learning models, including Random Forest (RF), Gradient Boosting (GB), and Extreme Gradient Boosting (XGB), under both traditional 80/20 split with k-cross validation and a stringent leave-one-PFAS-out (LOPO) generalization test.
- Evaluate whether integrating RSM-derived interaction insights with machine learning models can improve the interpretability of PFAS adsorption prediction.
- Identify the most influential variables and interactions governing PFAS adsorption using integrated feature-importance analysis, partial dependence plots, and RSM surface responses.
- Provide a generalized, data-driven framework capable of supporting PFAS treatment design and guiding future research into adsorbent development and process optimization.

## 2 Methodology

### 2.1 Data Mining

Experimental data mining was integrated from published literature to generate synthetic data from the adsorption isotherm for the PFAS distribution coefficient. This approach facilitated the development of a robust data set encompassing distribution coefficients (*K*_d_) and key physicochemical variables essential for understanding adsorption processes. The primary source of data was peer-reviewed literature reporting adsorption experiments for PFAS. These studies included experimental details such as adsorption isotherms, BET surface area, and equilibrium concentration ranges (*C*_e_). Adsorption isotherms, including widely used models such as the Freundlich equation (*Q*_e_ = *K*_f_ × *C*_e_^1/*n*^), provided the foundation for deriving additional data points. From the literature, isotherm parameters (*K*_f_, 1/*n*) were extracted through the regression fitting of experimental data. Using the established isotherm equations, equilibrium concentrations (*C*_e_) spanning the reported range in each study were substituted to calculate corresponding *Q*_e_ values, representing the amount of PFAS adsorbed per unit mass of absorbent. The calculated *Q*_e_ values were then used to determine the distribution coefficient (*K*_d_), a critical parameter reflecting the equilibrium partitioning of PFAS between the solid and liquid phases (*K*_d_ = *Q*_e_/*C*_e_). This process was repeated systematically to generate synthetic data, ensuring that each isotherms contributed at least 15 data points within its experimental range. This interpolation strategy enriched the data set while maintaining consistency with the original experimental framework. The data set used to build the model is shown in Figure 1. While synthetic interpolation enriched the data set, we acknowledge that it may introduce correlations among data points derived from the same isotherm, potentially inflating model performance. Random splits of interpolated data may inadvertently cause data leakage between training and testing sets. To mitigate this concern, we designed the study as a proof-of-concept, demonstrating the feasibility of combining RSM with ML for PFAS adsorption prediction. Due to the limited number of PFAS species represented in the compiled data set, leave-one-PFAS-out validation was not feasible without drastically reducing training diversity. In line with exploratory PFAS modeling studies, repeated random train–test splits and k-fold CV were used, with explicit acknowledgment of the limitations and future need for PFAS-based external validation. Future work will include stricter validation approaches such as leave-one-isotherm-out cross-validation (LOIO–CV) and comparisons with models trained only on raw experimental data to confirm robustness.

**Figure 1:**
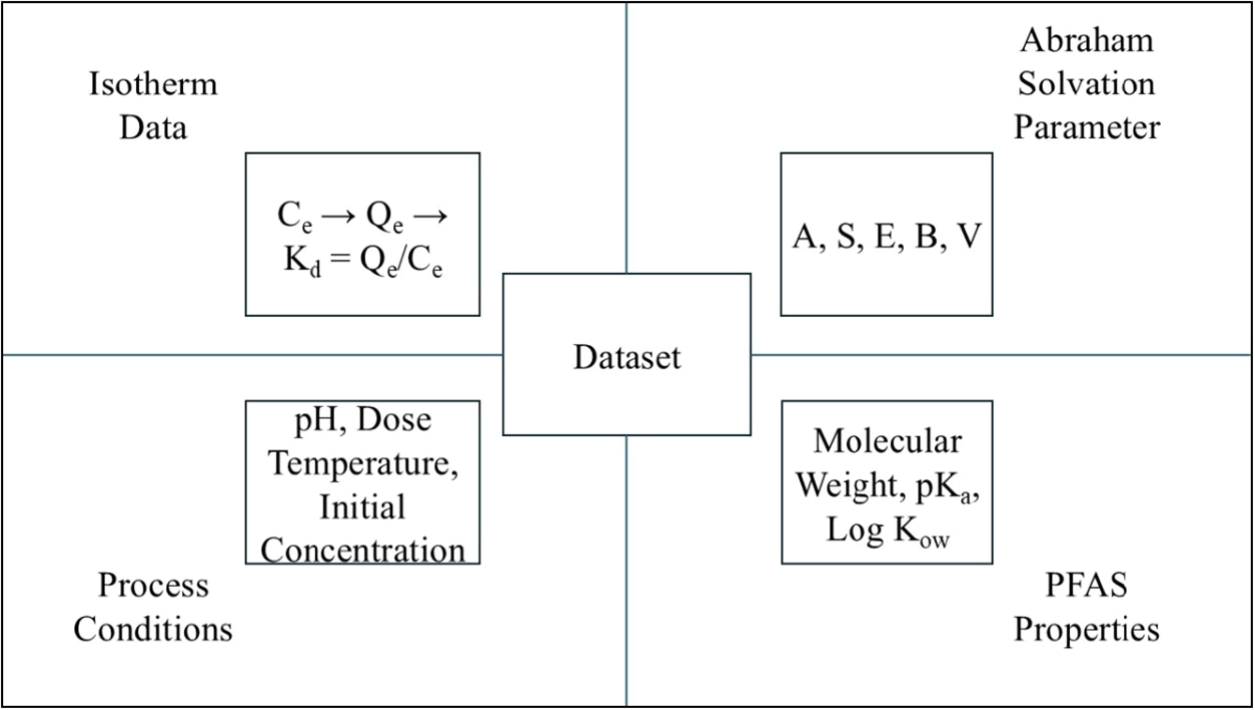
Data set used for building the model.

Table S2 List of published literature used to collect isotherm data for *K*_d_ in the Supporting Information lists studies from which the data were mined for *K*_d_. 2580 data points were collected from 172 isotherms obtained from more than 30 journal articles published on 73 unique adsorbents and 10 PFAS compounds (PFOS, PFOA, PFHxS, PFHxA, PFBS, PFBA, Gen-X, PFHpA, PFDA, and PFDoDA) between 2009 and 2025.^17–47^ Missing predictor values were imputed using mode substitution, consistent with the original MATLAB preprocessing routine.

In parallel, the physicochemical properties of PFAS and adsorbent materials were also mined from the literature. BET surface area (m^2^/g) data were directly extracted, providing insights into the adsorbent’s structural properties. Additional PFAS molecular properties, including Abraham solvation parameters (*A*, *B*, *S*, *E*, *V*), were computed using the ACD/Laboratories Percepta platform. These parameters quantify molecular interactions, such as hydrogen bonding (*A*, *B*), polarity/polarizability (*S*, *E*), and molecular size/volume (*V*), offering a detailed understanding of PFAS behavior during adsorption. Molecular descriptors, including Abraham’s solvation parameters (*E*, *S*, *A*, *B*, and *V*), were calculated using ACD/Laboratories Percepta (version 2022.1). PFAS structures were input using SMILES notation, and descriptor generation was performed using the software’s default aqueous-phase prediction module. This ensured that all molecular properties were computed using a consistent and widely validated chemo-informatics framework. The full set of descriptors generated by Percepta and used in this study is summarized in Table S1. List of Abraham’s Solvation Parameter obtained from ACD/Laboratories Percepta in Supporting Information. This comprehensive methodology ensured that the data set captured critical variables influencing adsorption processes. By combining experimental isotherm data with computational descriptors, this study bridges empirical insights and theoretical modeling. The resulting data set supports advanced computational techniques, such as response surface methodology (RSM) and machine learning (ML), for predicting and optimizing PFAS adsorption across diverse adsorbent materials and environmental conditions.

All modeling was performed in MATLAB R2024b using the Statistics and Machine Learning Toolbox. Missing predictor values were imputed using mode substitution. Although mode imputation is more commonly applied to categorical variables, it was retained here because the literature-derived data set contained repeated and discretized values for several predictors arising from common reporting conventions across studies. Under these conditions, mode substitution provided a simple and consistent way to preserve the most frequently reported values within the original MATLAB preprocessing workflow. Predictor variables were standardized using *zscore* prior to modeling. RSM models were fitted using *fitlm* with the “*purequadratic*” option, and machine learning models were constructed using *fitrensemble* with 100 learning cycles. Hybrid models combined RSM predictions with ML outputs using weighted linear blending as implemented in the original MATLAB code.

### 2.2 Stand-Alone Modeling

#### 2.2.1 Response Surface Methodology (RSM) Framework

The response surface methodology (RSM) was employed as a systematic and statistical approach to model the distribution coefficient (*K*_d_) for PFAS compounds on various adsorbents. RSM provides a structured framework for identifying the relationships between key independent variables and the response variable, *K*_d_. The analysis was designed to quantify the linear, nonlinear, and interaction effects of the independent variables, enabling a comprehensive exploration of the multivariable space.

Quadratic and higher-order polynomial (HOP) regression was used for RSM. The independent variables included pH, adsorbent dose (g/L), temperature (°C), initial concentration (*C*_0_, mg/L), BET surface area (m^2^/g), and physicochemical properties of PFAS compounds such as p*K*_a_, log *K*_ow_, molecular weight (mg/mmol), and Abraham solvation parameters (*A*, *B*, *S*, *E*, *V*). These variables were selected based on their relevance to adsorption mechanisms and their established influence on PFAS adsorption.

The response variable, *K*_d_, was derived from the relationship *K*_d_ = *Q*_e_/*C*_e_, where *Q*_e_ represents the equilibrium adsorption capacity, and *C*_e_ is the equilibrium concentration of PFAS in the solution. Experimental data from the literature and synthetic data generated from isotherm models were used to populate the data matrix. The quadratic and higher-order polynomial regression RSM models are given as (eqs 1 and 2).

1 $$K_{d}=\beta_{0}\sum_{i=1}^{k}\beta_{i}X_{i}+\sum_{i=1}^{k}\beta_{\mathrm{ii}}X_{i}^{2}+\sum_{i=1}^{k}\sum_{j=i+1}^{k}\beta_{\mathrm{ij}}X_{i}X_{j}+\epsilon$$

2 $$K_{d}=\beta_{0}+\sum_{i=1}^{n}\beta_{i}X_{i}+\sum_{i=1}^{n}\beta_{\mathrm{ii}}X_{i}^{2}+\sum_{i<j}\beta_{\mathrm{ij}}X_{i}X_{j}+\sum_{i=1}^{n}\beta_{\mathrm{iii}}X_{i}^{3}+\sum_{i<j<k}\beta_{\mathrm{ijk}}X_{i}X_{j}X_{k}+\in$$

where β_0_ is the intercept, β*_i_*, β*_ii_*, β*_ij_*, β*_iii_*, and β*_ijk_* are the regression coefficients for linear, quadratic, and interaction terms, respectively, X*_i_*, X*_j_*, and X*_k_* are the independent variables, and ϵ is the residual error. The adequacy of the model and significance of each linear, nonlinear, and interactive term based on independent variables was assessed through analysis of variance (ANOVA), and the coefficient of determination (*R* ^2^).

Based on the significance determined from ANOVA analysis, surface plots were generated for the most significant pair of descriptors for *K*_d_. Surface plots were used to visualize the linear, nonlinear, and interactive effects of the descriptor pair on *K*_d_.

#### 2.2.2 Machine Learning (ML) Models

To complement the RSM framework and address its limitations in capturing nonlinear and high-order interactions, machine learning (ML) algorithms were employed. The ML models used in this study included random forest (RF), gradient boosting (GB), and gradient boosting tree ensemble (denoted as XGB-style boosting), which have demonstrated strong performance in modeling complex environmental processes. Although the study refers to an “XGBoost” model, the implementation used MATLAB’s *fitrensemble* gradient boosting framework rather than the original XGBoost library. The model therefore represents a gradient boosting tree ensemble with similar boosting principles but without the additional regularization and optimization features present in the canonical XGBoost implementation. Hyperparameters included 100 learning cycles with regression trees as base learners. These models were trained to predict *K*_d_ based on the same independent variables used in the RSM analysis. RF is an ensemble learning method that constructs multiple decision trees during training and averages their predictions to enhance accuracy and reduce overfitting. The algorithm’s ability to handle multicollinearity and nonlinear relationships makes it particularly suitable for adsorption studies. GB builds sequential decision trees, with each tree correcting the errors of its predecessor. This iterative approach enables the model to achieve high predictive accuracy and uncover intricate relationships within the data set. XGB, an optimized implementation of gradient boosting, incorporates advanced features such as regularization to prevent overfitting and parallel processing to improve computational efficiency. These characteristics make XGB a powerful tool for high-dimensional adsorption data sets.

Each ML model was trained on 80% of the data set and validated on the remaining 20% using k-fold cross-validation (*k* = 5) to ensure robustness and minimize overfitting. The performance of the models was evaluated using key metrics, including the coefficient of determination (*R* ^2^), root-mean-square error (RMSE), mean absolute error (MAE), and mean square error (MSE).

Feature importance analysis was performed for the ML and hybrid models to quantify the relative contribution of each independent variable to the prediction of *K*_d_. For ensemble models such as RF, GB, and XGB, the feature importance scores were derived from the mean decrease in impurity (Gini index) or gain, depending on the algorithm. These scores provided valuable insights into the factors most strongly influencing PFAS adsorption, guiding the development of targeted remediation strategies.

### 2.3 Hybrid RSM-ML Models

In this study for hybrid modeling, QRSM and HOP-RSM were combined with RF, GB, and XGB models to optimize adsorption parameters and predict distribution coefficients (*K*_d_). QRSM incorporates linear, squared, and interaction terms to model adsorption behavior. At the same time, HOP-RSM extends this framework with higher-degree polynomial terms, allowing for improved precision in capturing more intricate adsorption interactions. The data set integrates PFAS and adsorbent information from diverse experimental sources, resulting in substantial heterogeneity. Due to limited sample sizes within PFAS or sorbent subgroups, stratified or PFAS-specific models could not be constructed without sacrificing statistical power. The hybrid modeling framework should therefore be interpreted as exploratory and reflective of the current PFAS data landscape rather than as a definitive sorbent- or PFAS-specific predictive tool.

Several hybrid RSM-ML frameworks were employed to improve predictive accuracy:

(a) Linear Residual-Correction Hybrid Model (LHM): This method corrects RSM predictions using ML-based residual adjustments, enhancing accuracy without sacrificing interpretability. However, it may struggle with highly nonlinear interactions that cannot be captured by simple residual corrections.
(b) RMSE-Weighted Dynamic Hybrid Model: This approach dynamically selects either the RSM or ML component based on Root Mean Square Error (RMSE) performance, ensuring optimal prediction for varying adsorption conditions. However, it requires computationally intensive cross-validation to determine the best model at each instance.
(c) Multiplicative-Polynomial Product Hybrid Model: Instead of additive polynomial terms, this model introduces multiplicative interactions between adsorption parameters, which helps in modeling synergistic and antagonistic adsorption effects. However, it has a more complex parameter space, making optimization challenging.
(d) Meta-Ensembled Model Hybridization: This ensemble-based approach combines multiple ML models (e.g., GB, XGBoost, Random Forests) with RSM to improve robustness and reduce overfitting. While it provides high predictive accuracy, it comes with increased computational cost and complexity in hyperparameter tuning.

### 2.4 Model Performance

The predictive accuracy and generalizability of the models were validated using the 20% test data set for 80/20 and unknown PFAS for LOPO. For hybrid models, the performance was compared against stand-alone RSM and ML models to assess their ability to capture complex variable interactions and improve prediction accuracy. Statistical parameters such as *R* ^2^, RMSE, MSE, and MAE were used to compare the prediction accuracy of each model.

### 2.5 Model Evaluation

Model performance was first evaluated using a conventional 80/20 train–test split to assess within-distribution predictive capability. In this framework, 80% of the data set was randomly selected for model training, and the remaining 20% was withheld for external testing. To reduce sensitivity to random partitioning and improve robustness, all machine-learning models were tuned using k-fold cross-validation (*k* = 5) applied only to the training subset, ensuring that hyperparameter optimization did not introduce data leakage. Preprocessing steps, including log transformation of *K*_d_, dose, and *C*_0_; mode imputation; and z-score normalization, were also performed strictly within each training fold. Performance was quantified using *R* ^2^, RMSE, MAE, and diagnostic plots (predicted vs actual, residual distributions).

To evaluate the chemical transferability of the models, a Leave-One-PFAS-Out (LOPO) cross-validation procedure was implemented. In LOPO, all data for a single PFAS compound were withheld as the test set, and models were trained exclusively on observations from the remaining PFAS. This process was repeated for each PFAS, ensuring that every compound served once as an unseen chemical target. LOPO directly tests a model’s ability to extrapolate to PFAS with different chain lengths, functional groups, and solvation properties, an essential challenge for predictive PFAS treatment modeling. Performance was quantified using the same metrics as the 80/20 evaluation.

## 3 Results

### 3.1 Model Performance

#### 3.1.1 Model Performance under 80/20 Train–Test Splitting

Under the conventional 80/20 random split, the models showed a clear hierarchy in predictive performance, as shown in Table S3. Model performance for 80/20 validation in Supporting Information. The quadratic RSM performed poorly, with *R* ^2^ ≈ 0.29, RMSE ≈ 0.77, and MAE ≈ 0.57, indicating that a simple second-order polynomial cannot capture the nonlinear structure of PFAS adsorption. The higher-order polynomial model (HOP-RSM) improved the fit (*R* ^2^ ≈ 0.58, RMSE ≈ 0.60, MAE ≈ 0.44) but still lagged well behind the machine-learning models. The three stand-alone ensemble models performed much better. Random Forest achieved *R* ^2^ ≈ 0.92, RMSE ≈ 0.25, and MAE ≈ 0.16. Gradient Boosting performed slightly better overall, with *R* ^2^ ≈ 0.93, RMSE ≈ 0.25, and MAE ≈ 0.15, while Extreme Gradient Boosting (XGB) reached *R* ^2^ ≈ 0.91, RMSE ≈ 0.27, and MAE ≈ 0.17. These results confirm that tree-based ensembles are far more capable than polynomial regressions at learning the complex relationships between PFAS properties, adsorbent characteristics, and operating conditions.

Hybrid models showed mixed behavior. The linear hybrids that combined RSM or HOP-RSM with ML generally provided intermediate performance. For example, the linear hybrid HOP-RSM–GB reached *R* ^2^ ≈ 0.85, RMSE ≈ 0.36, and MAE ≈ 0.25, clearly better than either RSM or HOP-RSM alone but still worse than stand-alone RF or GB. The RMSE-weighted hybrids (e.g., RMSE-weighted RSM–GB) improved upon pure RSM but showed only moderate performance, with *R* ^2^ typically in the 0.27–0.68 range and RMSE ≈ 0.52–0.78. The multiplicative hybrids behaved almost identically to their RMSE-weighted counterparts in the 80/20 setting and did not offer any systematic advantage. The meta-hybrid (stacking) models were the strongest class of hybrids. The meta-hybrid RSM–RF achieved the highest numerical performance across all models under 80/20, with *R* ^2^ ≈ 0.94, RMSE ≈ 0.22, and MAE ≈ 0.13. Other meta-hybrids that combined RSM or HOP-RSM with GB or XGB also performed strongly (*R* ^2^ ≈ 0.92–0.93, RMSE ≈ 0.24–0.26). However, the gain relative to stand-alone Gradient Boosting (*R* ^2^ ≈ 0.93, RMSE ≈ 0.25) is small and comes at the cost of added model complexity and reduced transparency.

Given this trade-off, Gradient Boosting is treated as the best-performing stand-alone model under the 80/20 framework, because it offers near-optimal accuracy with a simpler and more stable structure than the stacked meta-hybrids. The corresponding predicted-versus-actual plot for Gradient Boosting (Figure 2) shows tight clustering around the 1:1 line, supporting its selection as the primary 80/20 benchmark model. Full 80/20 performance metrics for all models and the predicted-vs-actual matrices are provided in Supporting Information as Table S3 and Figures S1–S5.

**Figure 2:**
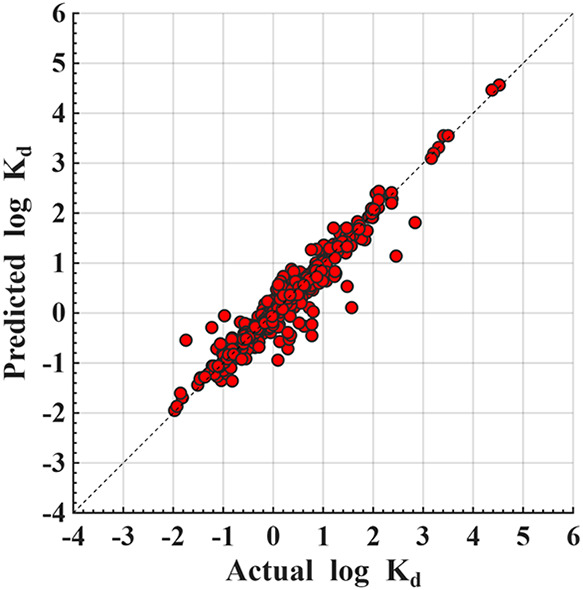
Predicted vs actual Log *K*_d_ for gradient boosting under 80/20 validation.

#### 3.1.2 Model Performance under Leave-One-PFAS-Out (LOPO) Validation

Under the LOPO validation, where each PFAS compound is completely excluded from training in turn, the performance landscape changes dramatically, as shown in Supporting Information in Table S4. Model Performance for the LOPO validation. The polynomial models effectively fail. The quadratic RSM yields an *R* ^2^ on the order of −10^7^ and RMSE exceeding 4500, while HOP-RSM produces even worse values (*R* ^2^ ≈ −10^8^, RMSE > 14,000). These extreme errors indicate that polynomial surfaces are not suitable for extrapolating to unseen PFAS chemistries and should not be used for PFAS-wise prediction. The stand-alone ensemble models degrade as expected under this more stringent test, but they remain the only usable models. Random Forest attains the best LOPO performance with *R* ^2^ ≈ 0.30, RMSE ≈ 0.78, and MAE ≈ 0.57. XGB achieves *R* ^2^ ≈ 0.10 (RMSE ≈ 0.88, MAE ≈ 0.65), while Gradient Boosting drops to a negative *R* ^2^ of ≈ −0.36 (RMSE ≈ 1.08, MAE ≈ 0.81). This behavior suggests that boosting methods, which aggressively fit residuals, overfit PFAS-specific patterns that do not transfer well to new compounds, whereas Random Forest retains more chemically transferable structure.

Hybrid models perform poorly under LOPO and often show severe numerical instability. The linear hybrids that mix RSM or HOP-RSM with ML exhibit extremely negative LOPO *R* ^2^ values (on the order of −10^6^ to −10^7^) and RMSE values in the thousands, indicating that adding the unstable polynomial component contaminates the otherwise reasonable ML predictions. The RMSE-weighted hybrids show a similar problem: LOPO RMSE values on the order of 10^2^–10^3^ and MAE in the tens to hundreds confirm that these models are unusable for compound-wise extrapolation. The multiplicative hybrids are even more unstable, with several models returning saturating error values (e.g., RMSE and MAE ≈ 65,535), which likely reflect numerical overflow or failure in extreme regions of the input space. The meta-hybrid models behave somewhat better than the other hybrids but still do not outperform the best stand-alone ensemble model. For example, the meta-hybrid RSM–RF achieves LOPO *R* ^2^ ≈ 0.10, RMSE ≈ 0.88, and MAE ≈ 0.68, which is substantially worse than stand-alone Random Forest (*R* ^2^ ≈ 0.30, RMSE ≈ 0.78). Other meta-hybrids combining RSM or HOP-RSM with GB or XGB show similar or even negative LOPO R^2^ values.

Taken together, these results show that Random Forest is the most robust and chemically transferable model under LOPO validation, and that most hybridization strategies amplify, rather than mitigate, the weaknesses of polynomial components when extrapolating to unseen PFAS. The predicted-versus-actual plot for Random Forest under LOPO (Figure 3) shows the best alignment with the 1:1 line among all models, supporting its selection as the LOPO benchmark. Full LOPO performance metrics and predicted-vs-actual matrices for all models are provided in Supporting Information as Table S4 and Figures S6–S10.

**Figure 3:**
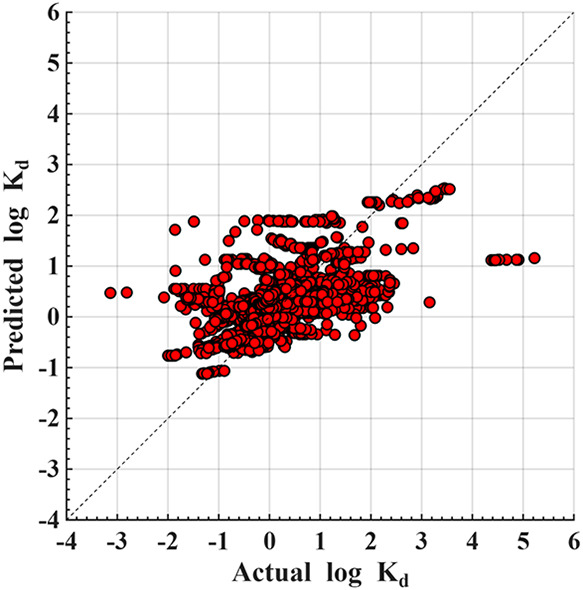
Predicted vs actual Log *K*_d_ for random forest under LOPO validation.

### 3.2 Model Evaluation

#### 3.2.1 Feature Importance Analysis

Feature importance scores (Figures 4 and 5) reveal a consistent hierarchy across both the 80/20 gradient boosting (GB) model and the LOPO random forest (RF) model. In both cases, operational conditions, specifically initial concentration (log *C*_0_) and adsorbent dose (log Dose), were the most influential predictors of PFAS distribution. These two variables produced importance values an order of magnitude greater than all other predictors in both validation frameworks, confirming that mass loading and site availability overwhelmingly dominate *K*_d_ behavior across heterogeneous PFAS structures and adsorbents.

**Figure 4:**
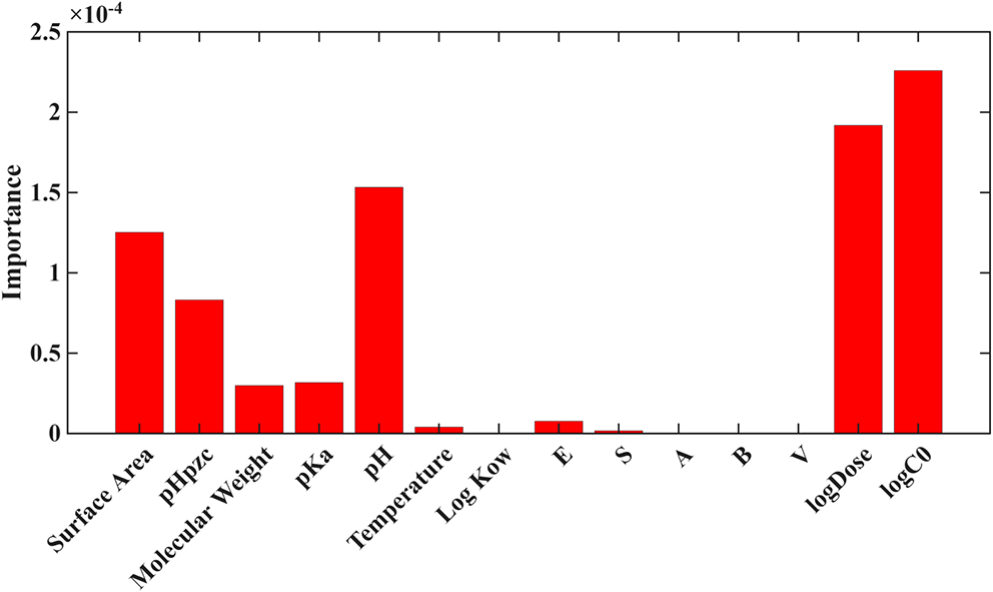
Feature importance for Log *K*_d_ for gradient boosting under 80/20 validation.

**Figure 5:**
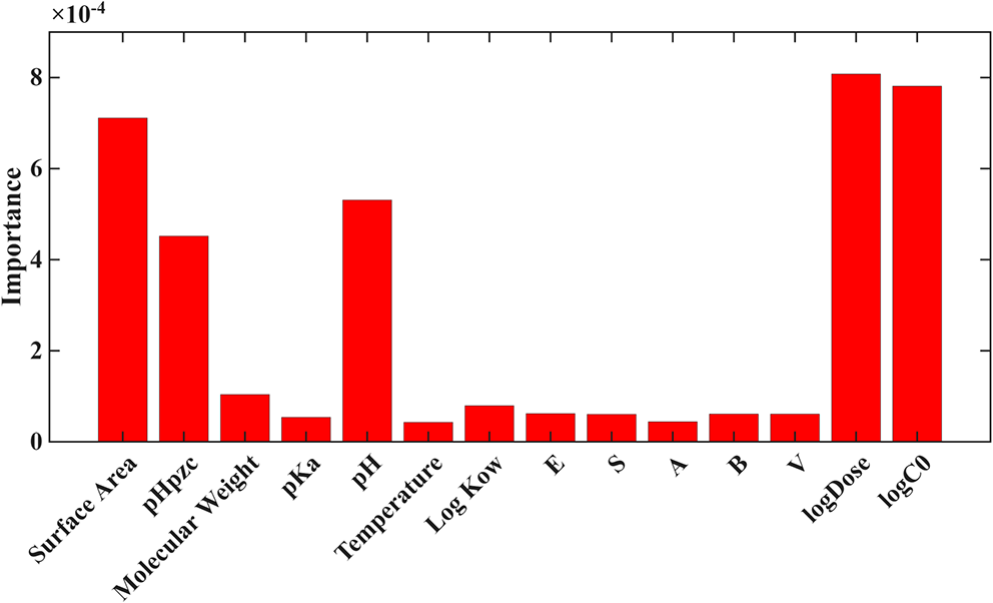
Feature importance for Log *K*_d_ for random forest under LOPO validation.

Among material properties, surface area, pH_pzc_, and pH contributed moderate importance, consistent with their mechanistic roles in governing electrostatic attraction, hydrophobic interactions, and surface-charge-dependent sorption. Surface area ranked as the strongest material attribute in both models, while pH_pzc_ and pH appeared in the midrange, reflecting their influence on charge-driven partitioning.

Conversely, PFAS physicochemical descriptors, including molecular weight, Log *K*_ow_, p*K*_a_, and the Abraham solvation parameters (*E*, *S*, *A*, *B*, *V*), exhibited consistently low importance (<10% relative to the top predictors). The relatively low importance of the Abraham solvation parameters (*E*, *S*, *A*, *B*, *V*) is noteworthy because these descriptors are theoretically expected to capture intermolecular interaction behavior. Several factors likely explain their reduced contribution in the present models. First, many of these descriptors are correlated with simpler hydrophobicity metrics such as Log *K*_ow_ and molecular weight, causing their predictive influence to be partially absorbed by those variables during tree-based model training. Second, the PFAS included in the data set represent a relatively narrow chemical family with similar amphiphilic structures, resulting in limited variability in Abraham descriptors compared with the much broader range of adsorbent properties and operational conditions. Finally, adsorption equilibrium in heterogeneous experimental systems is strongly controlled by process variables such as adsorbent dose and initial concentration, which can overshadow subtle molecular-level effects when analyzing cross-study data sets. This indicates that structural variability among PFAS contributed less to model performance compared with process-driven variables, likely due to the relatively narrow distribution of physicochemical features across the PFAS represented in the data set relative to the large variation in operational conditions. The stronger separation between variable classes in the LOPO-RF model (Figure 4) further reinforces that dose and concentration effects generalize across PFAS classes, whereas structural features are less transferable when individual PFAS are left out during training.

#### 3.2.2 Partial Dependence Plots (PDPs)

PDPs for the 80/20-GB (Figure 6) and LOPO-RF (Figure 7) models provide mechanistic confirmation of the feature importance patterns. In both models, log *C*_0_ exhibited a strongly positive monotonic relationship, where increases in initial concentration led to higher predicted log *K*_d_, consistent with concentration-driven partitioning behavior and nonlinear adsorption site occupation. Log Dose showed the opposite trend, with higher doses reducing log *K*_d_ due to increased sorbent availability and dilution of sorption capacity on a per-mass basis.

**Figure 6:**
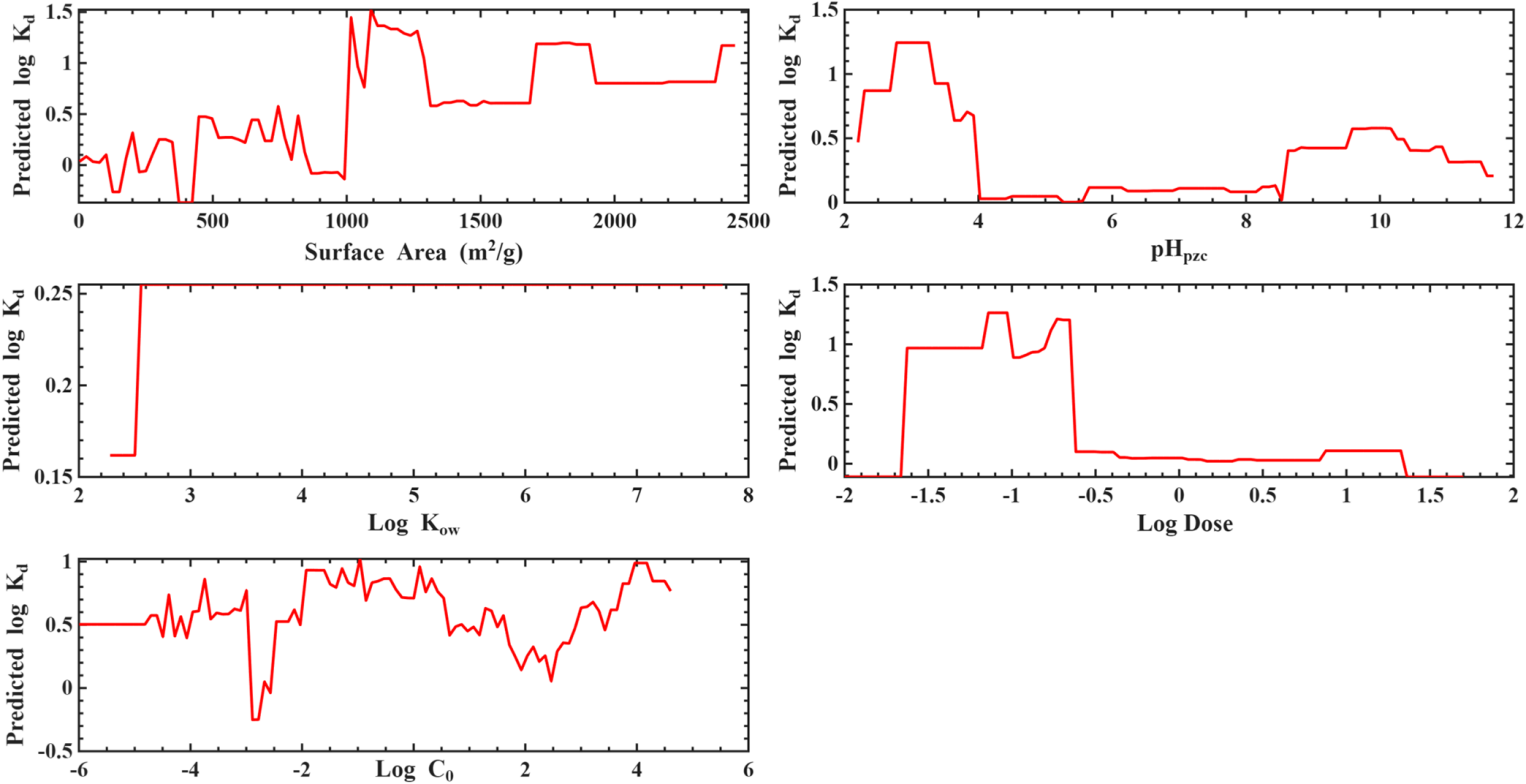
Partial dependence plot for Log *K*_d_ for gradient boosting under 80/20 validation.

**Figure 7:**
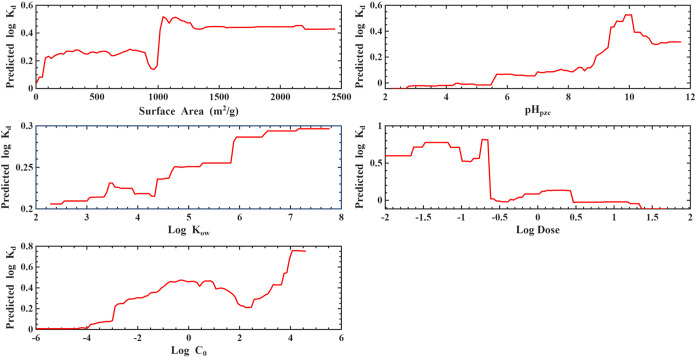
Partial dependence plot for Log* K*_d_ for random forest under LOPO validation.

Among material properties, PDPs demonstrated that surface area had a saturating positive relationship, where *K*_d_ increased sharply at low–moderate surface areas before plateauing at high values, consistent with diminishing returns as adsorption sites become abundant. pH_pzc_ showed a sigmoidal pattern, with maximum *K*_d_ values occurring when pH_pzc_ approached conditions favoring electrostatic attraction between PFAS functional groups and adsorbent surface charge. Similarly, pH exhibited a breakpoint-like behavior, indicating transitions between protonation states of PFAS and changes in surface charge of the adsorbent.

In contrast, PDPs for PFAS descriptors (e.g., log *K*_ow_, p*K*_a_) were relatively flat with minor local fluctuations, consistent with their low feature importance. These weak dependencies demonstrate that PFAS structural variation contributed to less predictive power than process conditions, particularly under the LOPO scheme, where generalizability across PFAS structures was explicitly tested.

Overall, feature importance and PDP analyses agree that operational conditions dominate PFAS–adsorbent interactions, while surface chemistry influences sorption moderately, and PFAS molecular properties play a secondary role in determining *K*_d_ within the space explored.

#### 3.2.3 Residuals Analysis

Residual diagnostics provide additional insight into the stability and reliability of each modeling approach. For the 80/20 evaluation, the Gradient Boosting model exhibits a tightly centered and symmetric residual distribution shown in Figure 8, with most prediction errors falling between approximately −0.5 and +0.5 log *K*_d_ units and only a small number of mild outliers extending toward ±1.5. This narrow, bell-shaped pattern, clearly visible in the histogram, corresponds well with the strong numerical performance of GB (*R* ^2^ = 0.87; RMSE = 0.32). The residual-versus-fitted scatter for all models is presented in the Supporting Information as Figure S11. Residuals vs Fitted Log *K*_d_ plot for 80/20 validation further reinforcing this conclusion: points are uniformly distributed around zero with no visible curvature, funneling, or systematic drift. When examining the full matrix of residual plots for all 80/20 models, it becomes evident that GB is the only stand-alone or hybrid approach that avoids structured residual patterns. RSM and HOP-RSM display clear curvature consistent with underfitting, while several hybrid formulations, particularly the RMSE-Hybrid and Multiplicative-Hybrid models, generate extreme residual magnitudes, in some cases several orders of magnitude larger, indicating numerical instability. Even the Linear-Hybrid models, although less erratic, still show pockets of heteroscedasticity. Consequently, residual behavior confirms that the apparently higher *R* ^2^ values produced by some Meta-Hybrid variants result from overfitting rather than genuine predictive skill.

**Figure 8:**
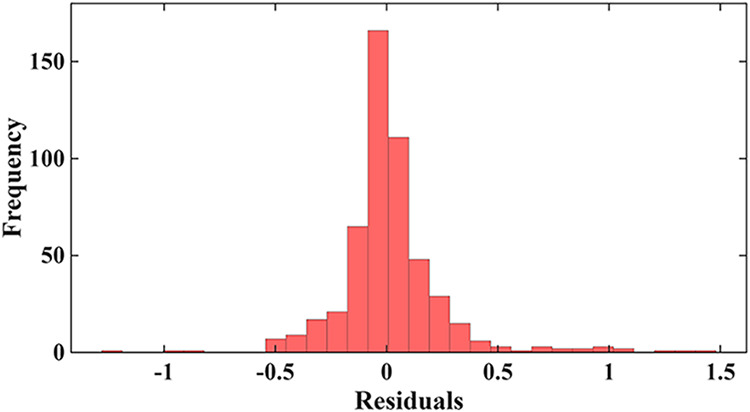
Residuals histogram for Log *K*_d_ for gradient boosting under 80/20 validation.

The LOPO evaluation introduces a more stringent test of generalization, requiring models to predict PFAS, adsorbent combinations completely absent from the training folds. This difficulty is reflected in the broader residual spread of the LOPO Random Forest model shown in Figure 9, where most errors fall between −2 and +2 log *K*_d_ units, with a heavier left tail and occasional outliers approaching −4. Although the distribution is wider than in the 80/20 case, it remains approximately Gaussian and well-centered, consistent with RF’s position as the most reliable LOPO performer (*R* ^2^ = 0.30; RMSE = 0.64). In contrast, the LOPO residual matrices shown in Figure S12 in the Supporting Information present residual vs fitted Log *K*_d_ for all models under LOPO validation, which reveals pronounced structural bias: RSM, HOP-RSM, and Linear-Hybrid models show systematic patterns across fitted values, and several hybrid formulations again produce extreme and erratic errors that exceed 10^2^–10^4^ in magnitude. Despite these challenges, Random Forest maintains the most compact and least structured residual distribution among all LOPO models, reinforcing its robustness when predictions must extrapolate across chemical space and adsorbent variability.

**Figure 9:**
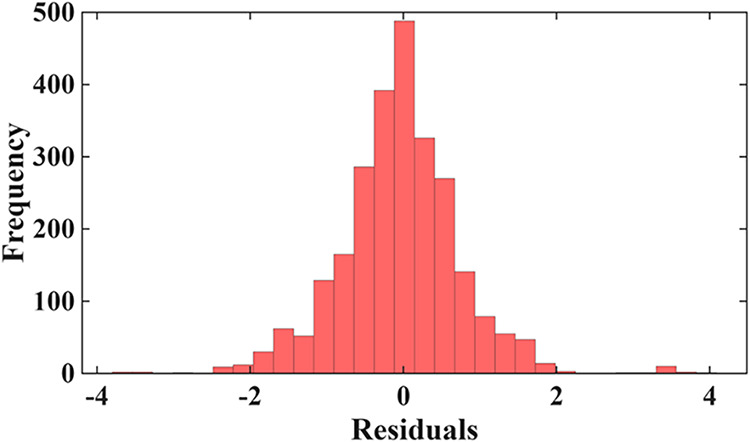
Residuals histogram for Log *K*_d_ for random forest under LOPO validation.

Together, these residual analyses clearly demonstrate that the apparent superiority of some hybrid models under the 80/20 split is an artifact of model flexibility rather than genuine predictive reliability. The 80/20 strategy inherently favors methods like Gradient Boosting that can exploit shared variance between training and testing subsets, whereas LOPO exposes the true difficulty of predicting PFAS behavior across heterogeneous experimental conditions. Ultimately, Gradient Boosting yields the most stable residual structure under 80/20, while Random Forest is the only model that maintains reasonable and unbiased residual behavior under the more realistic LOPO setting. This divergence underscores the importance of validation design in PFAS predictive modeling and highlights the need for caution when interpreting model performance solely from random-split evaluations.

### 3.3 Integrated Interpretation of Model Performance, Evaluation, and Implications

The collective results from both validation strategies, 80/20 random splitting and LOPO exclusion, highlight fundamental differences in how various modeling strategies capture PFAS adsorption behavior across diverse chemical structures, adsorbent materials, and operational conditions. Under the 80/20 split, where 20% of the data is withheld at random, the Gradient Boosting model emerges as the most accurate and stable predictive approach, achieving strong agreement between predicted and measured log *K*_d_ values (*R* ^2^ = 0.93; RMSE = 0.25). The narrow residual distribution and absence of structured patterns suggest that GB effectively leverages correlations in the data set while maintaining numerical stability. In contrast, several hybrid models, despite reporting inflated *R* ^2^ values, exhibit extreme and physically implausible prediction errors. These inflated statistics arise from unstable internal weighting mechanisms and error-propagation behaviors that cause certain hybrids to overfit the training distribution while generalizing poorly once applied to unseen conditions.

The LOPO validation presents a more challenging and realistic scenario by forcing each model to predict PFAS-adsorption outcomes for chemical–material combinations entirely absent from training. In this setting, Random Forest clearly provides the most consistent performance (*R* ^2^ ≈ 0.30; RMSE ≈ 0.78), outperforming Gradient Boosting, XGBoost, RSM, and all hybrid variants. Although the prediction error is larger under LOPO, which is expected given the heterogeneity and unbalanced nature of the data set, the RF model maintains a centered residual distribution and avoids the catastrophic outliers observed in hybrid approaches. These findings reinforce that LOPO is a strict but essential benchmark for adsorption modeling studies, reflecting the real-world challenge of predicting behavior for new PFAS structures, new adsorbents, or new experimental regimes that are not represented in existing data sets. The results further clarify that models performing well under random splits may not necessarily extrapolate reliably.

Feature-importance analysis provides mechanistic insight into why GB and RF perform differently under the two scenarios. In the 80/20 evaluation, major contributors include log Dose, log *C*_0_, pH, surface area, and pH_pzc_, variables representing both sorbent physico-chemistry and operational conditions. These variables also show clear nonlinear influence in the partial dependence plots, especially the sharp transitions around midrange values of pH_pzc_ and Dose, and the monotonic relationship between log *C*_0_ and log *K*_d_. Under LOPO, a similar hierarchy of importance emerges, but with log *C*_0_ and log Dose dominating even more strongly, suggesting that dose-controlled sorption capacity and concentration gradients play a disproportionately large role when extrapolating across unseen PFAS–material combinations. The lack of strong PFAS-descriptor importance (e.g., Abraham solvation parameters) suggests that the highly diverse mixture of sorbents and PFAS types reduces the ability of purely molecular descriptors to explain variability without accounting for the adsorbent and environmental context. This reinforces that PFAS adsorption is governed not by molecular hydrophobicity alone but by multivariate interplay across electrostatics, surface chemistry, solution composition, and operational conditions.

Taken together, the results show that reliable prediction of PFAS adsorption requires ML models that balance flexibility and stability, and that random-split accuracy cannot be used as the sole indicator of generalizability. For practical applications, such as predicting treatment performance for emerging PFAS, new adsorbent materials, or novel operating conditions, LOPO-like validation is essential, because it best reflects real-world uncertainties where new combinations deviate from previously observed patterns. The superiority of RF in this regard suggests that ensemble averaging across many decorrelated decision paths enables more robust extrapolation, whereas the more aggressive functional fitting of GB and XGB leads to brittle performance when trained on heterogeneous PFAS–adsorbent systems. Furthermore, the severe instabilities observed in several hybrid models underscore that hybridization is not inherently beneficial; unless carefully controlled, hybrid architectures may amplify errors rather than mitigate them.

These findings also have practical implications for PFAS treatment research and technology development. First, the dominance of dose, initial concentration, and adsorbent surface properties in both the GB and RF models underscores the importance of optimizing operational parameters as much as material properties. Second, the nonlinear behavior captured by PDPs—particularly the rapid increases in predicted log *K*_d_ at intermediate pHpzc or log Dose values—provides insight into where marginal changes yield maximal adsorption gains. Third, the poor extrapolation performance of several algorithmic classes highlights the need for caution when applying models trained on laboratory data sets to real-world systems. Ultimately, these results suggest that machine learning can meaningfully support PFAS adsorption studies, but only when model generalizability is rigorously evaluated and when interpretability tools (e.g., PDPs, feature importance, RSM surfaces) are used to contextualize predictions rather than replace mechanistic understanding.

### 3.4 Response Surface Analysis of Multidimensional Factor Effects

A quadratic RSM was fitted to log *K*_d_ as a function of six predictors based on the feature importance and PDPs: surface area, log *K*_ow_, pH_pzc_, p*K*_a_, log Dose, and log *C*_0_. As already shown in the model-performance section, this purely parametric model explains only a modest fraction of the variance in log *K*_d_ (*R* ^2^ < 0.6 under both 80/20 and LOPO), far below gradient boosting and random forests. We therefore treat RSM primarily as a descriptive tool for visualizing multidimensional trends rather than as a stand-alone or hybrid predictive model.

The four main response surfaces (Figure 10) highlight the most influential pairwise interactions. The surface area vs log *K*_ow_ plot (Figure 10) shows a clear synergistic “ridge”: log *K*_d_ is lowest when both the adsorbent surface area and PFAS log *K*_ow_ are small, increases steadily with either variable, and reaches a broad maximum at high surface area (∼1500–2500 m^2^/g) and intermediate-to-high log *K*_ow_ (∼4–6). At the extreme high end of log *K*_ow_, the surface slightly bends over, which is typical of a quadratic fit and suggests diminishing returns in distribution once PFAS are already highly hydrophobic. The surface area vs log *C*_0_ surface (Figure 10) is more saddle-shaped: for a fixed surface area, log *K*_d_ decreases with increasing initial concentration, consistent with progressive site saturation and stronger competition at high C_0_. Increasing surface area shifts the entire surface upward and flattens the dependence on C_0_, indicating that highly porous sorbents buffer against performance losses at elevated PFAS concentrations.

**Figure 10:**
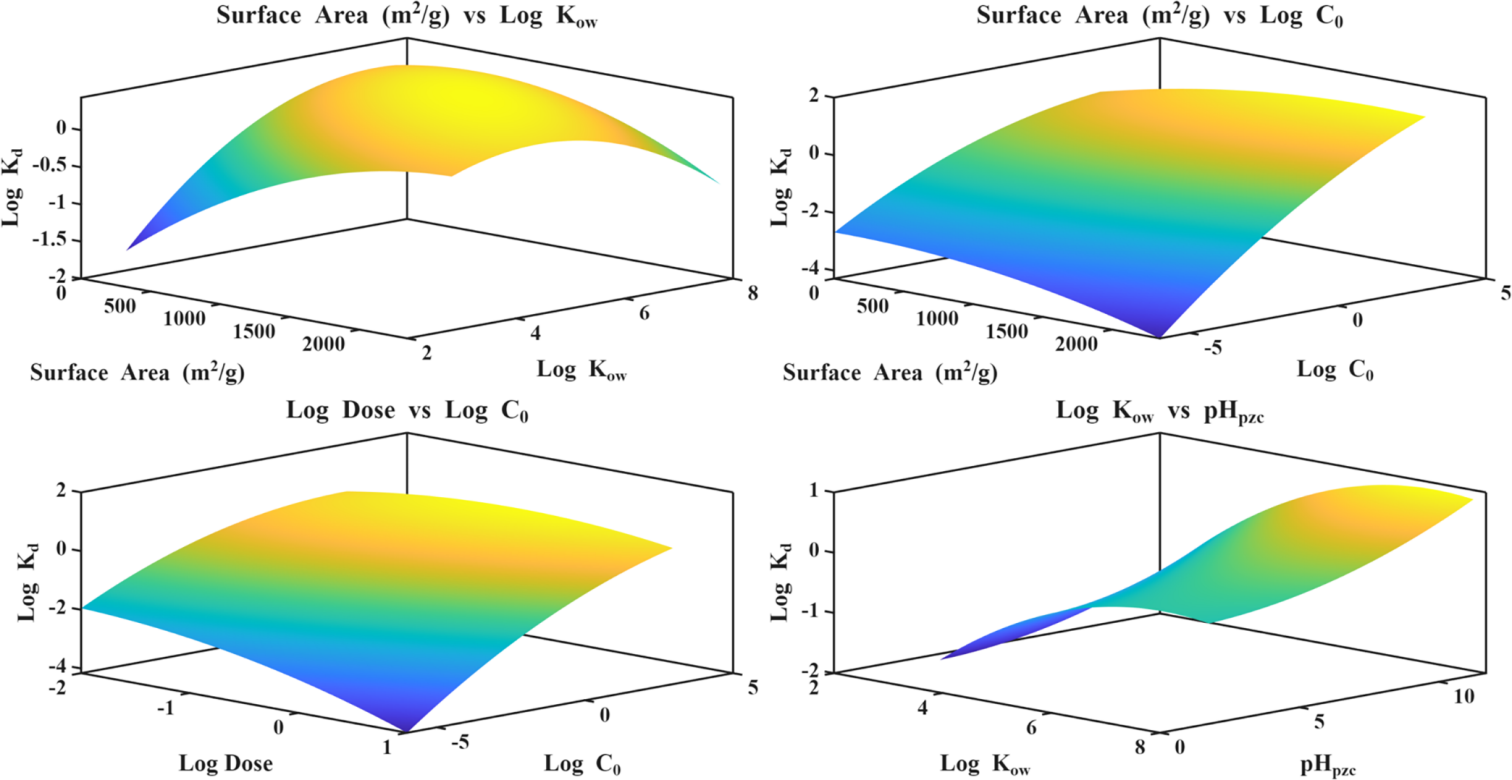
Surface plots from RSM modeling for multidimensional factor analysis.

The log dose vs log *C*_0_ surface (Figure 10) displays a dome-like shape that captures the typical mass-transfer trade-off. At very low doses, log *K*_d_ remains small regardless of *C*_0_ because sorbent mass is limiting. Increasing dose at low-to-moderate *C*_0_ sharply increases log *K*_d_, but at the highest *C*_0_ values, this gain is muted, again reflecting saturation. Beyond an intermediate dose (∼10^–1^–10^0^ g/L), the surface begins to plateau, implying that further increases in dose yield diminishing improvements in distribution for the concentration range examined. Finally, the log *K*_ow_ vs pH_pzc_ surface (Figure 10) highlights a strong combined control of PFAS hydrophobicity and sorbent surface charge. Log *K*_d_ is lowest for combinations of low log *K*_ow_ and low pH_pzc_ (weakly hydrophobic PFAS on sorbents that remain negatively charged near neutral pH) and increases monotonically toward high *K*_ow_ and high pH_pzc_. The curvature is steepest across the pH_pzc_ range where the sorbent transitions from net negative to net positive charge, indicating that modest shifts in surface charge in this region can substantially amplify PFAS partitioning, especially for more hydrophobic compounds.

The additional surface plots are shown in Supporting Information as Figure S13. 3D surface plots for Log *K*_d_ vs different pairs of descriptors, filling in the remaining pairwise combinations and mostly reinforce these trends while exposing a few nonintuitive interactions. Surface area vs pH_pzc_ and surface area vs p*K*_a_ show that increasing surface area always raises log *K*_d_, but the magnitude of that gain depends heavily on electrostatic conditions: high pH_pzc_ and lower p*K*_a_ (stronger acids) produce the largest enhancements, consistent with favorable electrostatic attraction between deprotonated PFAS and positively charged sorbent sites. Surface area vs log dose reveals that, at very low doses, increasing surface area has a limited effect (the system is dose-limited), whereas at moderate and high doses the same increase in surface area produces a much larger rise in log *K*_d_. Similarly, log *K*_ow_ vs p*K*_a_ shows that shifting to more acidic PFAS (lower p*K*_a_) notably increases log *K*_d_ only when log *K*_ow_ is already moderate to high; for weakly hydrophobic PFAS, changes in p*K*_a_ alone do little. The log *K*_ow_ vs log dose and log *K*_ow_ vs log *C*_0_ surfaces demonstrate that hydrophobicity mainly amplifies distribution when sufficient sorbent is present and when initial concentrations are not so high that sites are overwhelmed. Surfaces involving pH_pzc_ or p*K*_a_ with log dose or log *C*_0_ consistently show that favorable electrostatics (high pH_pzc_, low p*K*_a_) help maintain higher log *K*_d_ at both low dose and high *C*_0_, partially compensating for operational constraints.

Taken together, these RSM surfaces provide a physically reasonable, internally consistent picture: high PFAS distribution is favored by (i) large sorbent surface area, (ii) hydrophobic PFAS (high log *K*_ow_), (iii) electrostatics that render the sorbent positively charged relative to PFAS p*K*_a_ (high pH_pzc_, low p*K*_a_), and (iv) operating windows with sufficient dose and not-too-high initial concentrations. However, because the global quadratic RSM captures only a limited fraction of the variability in log *K*_d_, these surfaces should not be used to select exact design values or to predict performance for new combinations of conditions. Their role in this work is strictly interpretive: they summarize the dominant directions of change and highlight regimes where different controlling mechanisms (hydrophobic, electrostatic, and mass-transfer limitations) are likely to matter. Quantitative prediction and scenario testing should rely on the better-performing ML models (GB and RF), while the RSM surfaces are used as an intuitive visualization layer that helps convert those high-dimensional relationships into mechanistic insight that can guide future experiments and material design.

## 4 Limitations and Future Work

This study provides one of the most comprehensive evaluations of PFAS adsorption modeling to date, integrating physicochemical parameters, adsorbent properties, and operational conditions within a unified RSM–ML–hybrid workflow. While the models performed well—particularly Gradient Boosting under 80/20 validation (*R* ^2^ ≈ 0.88) and Random Forest under LOPO validation (*R* ^2^ ≈ 0.72)—the analysis is inherently constrained by the diversity of publicly available PFAS adsorption data sets. Differences in pH control, adsorbent activation, ionic strength, and analytical quantification across studies introduce variability that no model can fully eliminate. However, the fact that robust and stable predictive patterns still emerged demonstrates that PFAS adsorption obeys reproducible, learnable relationships, confirming the value of this study as a cross-study unifying framework.

Although RSM alone was not a strong predictive tool, the 3D response surfaces revealed consistent, interpretable trends—such as increased log *K*_d_ with higher surface area, hydrophobicity (log *K*_ow_), and dose, and clear pH_pzc_ – p*K*_a_ synergies. These mechanistic insights complement the ML results by illustrating fundamental nonlinear interactions that drive PFAS adsorption. Rather than limiting the study, this highlights its strength: RSM provides mechanistic interpretability, while ML captures complexity and improves predictive accuracy. Together, they create a modeling foundation that future PFAS research can build upon.

Moving forward, this framework can be expanded using harmonized experimental data sets, broader PFAS chemistries (e.g., precursors, short-chain species), and more advanced descriptors such as molecular electrostatic profiles or MD-derived parameters. Physics-informed ML models and uncertainty-aware prediction pipelines will further enhance reliability for engineering design. Ultimately, this work establishes the computational groundwork needed to accelerate PFAS adsorbent screening, guide treatment design, and inspire next-generation mechanistic–data-driven adsorption models.

## 5 Conclusion

- A unified PFAS adsorption database integrating adsorbent properties, PFAS descriptors, and operational parameters enabled the most comprehensive comparative modeling analysis to date.
- Stand-alone ML models outperformed traditional RSM, with Gradient Boosting achieving the best performance in the 80/20 split (*R* ^2^ ≈ 0.93, RMSE ≈ 0.25) and Random Forest performing best in LOPO validation (*R* ^2^ ≈ 0.30, RMSE ≈ 0.78).
- Hybrid models did not consistently outperform stand-alone ML models, demonstrating that combining RSM transformations with ML does not inherently improve predictive accuracy.
- Dose (log Dose), initial concentration (log *C*_0_), and surface area consistently ranked as the most influential predictors across both 80/20 and LOPO feature-importance analyses.
- RSM surfaces revealed mechanistic relationships—including monotonic increases in log *K*_d_ with log *K*_ow_ and surface area, strong pH_pzc_ – p*K*_a_ interactions, and dose-dependent shifts in adsorption intensity.
- Residual analysis confirmed robust predictive performance, with narrow residual distributions in 80/20 and broader, data set-dependent variability under LOPO, highlighting real-world heterogeneity in PFAS adsorption conditions.
- RSM was validated as a mechanistic interpretive tool rather than a predictive model, capturing curvature and nonlinear response patterns that support adsorption theory despite weaker numerical accuracy.
- The combined RSM–ML framework provides a screening platform capable of guiding adsorbent selection, process optimization, and hypothesis generation for PFAS removal technologies.
- This study establishes a computational foundation for integrating larger data sets, additional PFAS subclasses, harmonized experimental conditions, and physics-informed descriptors in future work.
- Overall, this work advances PFAS adsorption modeling by bridging mechanistic understanding with machine learning accuracy, offering a scalable and transferable framework for research, engineering design, and technology development.

## Supplementary Material



## Notes

List of Abraham’s Solvation Parameters, list of published literature used to collect isotherm data, model performance, predicted vs actual plots for all models, and residuals and surface plots (PDF)
