# Supplementary material for: A Hybrid Response Surface Methodology and Machine Learning Framework for Quantifying Effects of Physicochemical Parameters on PFAS Distribution
Source: ACS ES T Water. 2026 Apr 17;6(5):2761–72. doi: 10.1021/acsestwater.5c01162 (PMC13162251; doi:10.1021/acsestwater.5c01162)
Supplement: Supplementary file 1 [file ew5c01162_si_001.pdf]

A Hybrid Response Surface Methodology and Machine Learning Framework for Quantifying Effects of Physicochemical Parameters on PFAS Distribution

Harsh V. Patel[1], Jazmin Green[2], Hyo-Shin (John) Park[3], Stephanie Luster-Teasley Pass[1], Renzun Zhao[1,*]

[1]Department of Civil, Architectural, and Environmental Engineering, North Carolina A&T State University, Greensboro, NC 27411, USA

[2]Department of Computer Science, North Carolina A&T State University, Greensboro, NC 27411, USA

[3]Department of Engineering Management and Systems Engineering, Old Dominion University, Norfolk, VA 23529, USA

*Corresponding author. Phone: (336) 285-3684; fax: (336)-334-7540; e-mail: rzhao@ncat.edu

Supporting Information

Table S1: List of Abraham's Solvation Parameters obtained from ACD/Labs Percepta

PFAS Compounds	Abraham's Solvation Parameter				
	E	S	A	B	V
**11Cl-PF3OUdS**	-1.1	0.35	0.31	0.9	2.3903
**3:3 FTCA**	-0.78	0.6	0.31	0.92	2.0381
**4:2 FTS**	-0.77	0.25	0.84	0.65	1.4979
**5:3 FTCA**	-0.56	0.04	1.01	0.51	1.2807
**6:2 FTS**	-0.68	0.37	0.84	0.58	1.1633
**7:3 FTCA**	-0.27	0.16	0.84	0.33	0.8698
**8:2 FTS**	-0.31	0.74	0.31	0.76	1.1702
**9Cl-PF3ONS**	-0.94	0.25	0.31	0.72	2.1564
**ADONA**	-1.21	-0.58	0.84	0.26	1.9264
**HFPO-DA/Gen-X**	-1.53	-0.83	0.84	0.24	2.2786
**NEtFOSA**	-0.42	0.87	0.31	0.89	1.2289

**NEtFOSAA**	-0.78	0.37	0.31	0.73	1.6985
**NEtFOSE**	-0.74	-0.21	0.84	0.3	1.3981
**NFDHA**	-0.31	0.75	0.31	0.77	1.452
**NMeFOSA**	-0.63	0.49	0.31	0.74	1.5224
**NMeFOSAA**	-0.59	-0.09	0.84	0.31	1.222
**NMeFOSE**	-0.46	0.35	0.84	0.43	0.9285
**PFBA**	-0.62	0.23	0.84	0.42	1.1046
**PFBS**	-1.06	-0.46	0.84	0.27	1.7503
**PFDA**	-0.63	0.5	0.31	0.74	1.8042
**PFDoA**	-0.94	0.24	0.31	0.71	1.8746
**PFDoS**	-0.9	-0.34	0.84	0.29	1.5742
**PFDS**	-0.43	0.04	0.84	0.32	1.0459
**PFEESA**	-0.47	0.61	0.31	0.75	1.3463
**PFHpA**	-1.37	-0.71	0.84	0.25	2.1025
**PFHpS**	-0.58	0.43	0.57	1.11	2.5537
**PFHxA**	-0.58	0.43	0.57	1.11	2.4128
**PFHxS**	-1.84	-1.08	0.84	0.22	2.6308
**PFMBA**	-1.69	-0.95	0.84	0.23	2.4547
**PFMPA**	-0.65	0.01	0.44	0.71	1.9157
**PFNA**	-0.76	-0.03	0.37	0.72	2.0566
**PFNS**	-0.76	-0.03	0.37	0.72	2.1975
**PFOA**	-1.1	0.12	0.31	0.7	2.0507
**PFOS**	-1.25	0	0.31	0.69	2.2268
**PFOSA**	-1.57	-0.25	0.31	0.67	2.579
**PFPeA**	-0.51	0.2	0.23	1.15	2.3971
**PFPeS**	-0.51	0.2	0.23	1.15	2.538
**PFTeDA**	-0.27	0.17	0.57	0.34	1.1516
**PFTrDA**	-0.59	-0.08	0.57	0.32	1.5038
**PFUnA**	0.9	-0.33	0.57	0.29	1.856

Table S2: List of published literature used to collect isotherm data for K_d.

**Adsorbent**	**PFAS**	**Study**
**PAC**	PFOS	[1]
**PAC**	PFOA	

**GAC**	PFOS	
**GAC**	PFOA	
**W400**	PFOS	
**M400**	PFOS	
**MA**	PFOS	
**SWCNT**	PFOS	2
**MWCNT10**	PFOS	
**MWCNT50**	PFOS	
**Bamboo GAC**	PFOS	
**Bamboo GAC**	PFOA	
**SCGKOH**	PFOS	
**F300**	PFOS	3
**MCG**	PFOS	
**GO-PEI3**	PFOA	4
**PEI-f-cellulose**	PFOA	5
**OMC-700**	PFOA	
**OMC-900**	PFOA	
**SWCNT**	PFOA	
**MWCNT-10**	PFOA	6
**MWCNT-20**	PFOA	
**MWCNT-40**	PFOA	
**MWCNT-100**	PFOA	
**SWCNT**	PFOS	
**SWCNT**	PFHxS	
**SWCNT**	PFHxA	7
**SWCNT**	PFBS	
**SWCNT**	PFBA	
**MWCNT**	PFOA	
**Fe-CNT**	PFOA	8
**Cu-CNT**	PFOA	
**Zn-CNT**	PFOA	
**Na-MT**	PFOS	
**0.2CEC-Mt**	PFOS	
**0.5CEC-Mt**	PFOS	9
**1.0CEC-Mt**	PFOS	
**2.5CEC-Mt**	PFOS	
**Magnetic Nanocomposite**	PFOS	10
**Magnetic Nanocomposite**	PFOA	
**Ammoniated Magnetic Particle**	PFOS	11
**Ammoniated Magnetic Particle**	PFOA	
**Calcined Hydrotalcite**	PFOA	12
**Calcined Hydrotalcite**	PFOS	13

**AI400**	PFOS	
**AI400**	PFOA	
**Chitosan**	PFOS	14
**Cyclodextrin Polymer 1**	PFOA	
**Cyclodextrin Polymer 1**	Gen-X	15
**Cyclodextrin Polymer 2**	PFOA	
**Cyclodextrin Polymer 2**	Gen-X	
**Filtrasorb 400**	PFOS	
**DowL493**	PFOS	16
**Amb XAD 4**	PFOS	
**DowV493**	PFOS	
**Polyacrylonitrile fiber (PANF)-derived activated carbon fibers (PACFs)**	PFOS	
**Polyacrylonitrile fiber (PANF)-derived activated carbon fibers (PACFs)**	PFOA	
**PAC**	PFOS	17
**PAC**	PFOA	
**GAC**	PFOS	
**GAC**	PFOA	
**agro-waste biomass of Vitis vinifera (grape) leaf litter - KOH**	PFOS	
**agro-waste biomass of Vitis vinifera (grape) leaf litter - KOH**	PFOA	18
**agro-waste biomass of Vitis vinifera (grape) leaf litter - H3PO$**	PFOS	
**agro-waste biomass of Vitis vinifera (grape) leaf litter - H3PO4**	PFOA	
**GAC**	PFOS	
**GAC**	PFOA	19
**GAC**	PFHPA	
**Magnetic Activated Carbon**	PFOS	
**Magnetic Activated Carbon**	PFOA	20
**Magnetic Activated Carbon**	PFBS	
**Magnetic Activated Carbon**	PFHxS	
**PAC**	PFOS	
**PAC**	PFOS	
**PAC**	PFOS	21
**PAC**	PFOS	
**PAC**	PFOS	
**PAC**	PFOS	
**1240C**	PFOS	
**1240C**	PFOA	22
**1240 HT**	PFOS	

**1240 HT**	PFOA	
**1240 AT**	PFOS	
**1240 AT**	PFOA	
**F400**	PFOS	
**F400**	PFOA	
**F400 HT**	PFOS	
**F400 HT**	PFOA	
**F400 AT**	PFOS	
**F400 AT**	PFOA	
**BioNC**	PFOS	
**BioNC**	PFOA	
**BioNC HT**	PFOS	
**BioNC HT**	PFOA	
**BioNC AT**	PFOS	
**BioNC AT**	PFOA	
**WVB**	PFOS	
**WVB**	PFOA	
**WVB HT**	PFOS	
**WVB HT**	PFOA	
**WVB AT**	PFOS	
**WVB AT**	PFOA	
**ACF20**	PFOS	
**ACF20**	PFOA	
**ACF20 AT**	PFOS	
**ACF20 AT**	PFOA	
**Covalent triazine-based framework**	PFOA	
**Covalent triazine-based framework**	PFOS	
**Covalent triazine-based framework**	PFHxA	
**Covalent triazine-based framework**	PFHxS	
**Covalent triazine-based framework**	PFBA	
**Covalent triazine-based framework**	PFBS	
**Calgon AC**	PFOA	23
**Calgon AC**	PFOS	
**Calgon AC**	PFHxA	
**Calgon AC**	PFHxS	
**Calgon AC**	PFBA	
**Calgon AC**	PFBS	
**single-walled carbon nanotube**	PFOA	
**single-walled carbon nanotube**	PFOS	
**Boehmite**	PFOS	24
**Boehmite**	PFOA	
**Alumina**	PFOA	25
**Alumina**	PFOS	

**Alumina NP**	PFOS	
**Alumina NP**	PFOS	
**Alumina NP**	PFOS	26
**Alumina NWs**	PFOS	
**Alumina NWs**	PFOS	
**Alumina NWs**	PFOS	
**Goethite**	PFOS	27
**MIL-96(Al)**	PFOA	28
**MIL-100(Al)**	PFOA	
**Montmorillonite**	PFOS	
**Montmorillonite**	PFOA	
**Montmorillonite**	PFHxA	
**Montmorillonite**	PFHxS	
**Kaolinite**	PFOS	
**Kaolinite**	PFOA	29
**Kaolinite**	PFHxA	
**Kaolinite**	PFHxS	
**Hematite**	PFOS	
**Hematite**	PFOA	
**Hematite**	PFHxA	
**Hematite**	PFHxS	
**Ferrihydrite**	PFOS	30
**Ferrihydrite**	PFOA	
**Coal Fly-Ash A**	PFBA	
**Coal Fly-Ash A**	PFHxA	
**Coal Fly-Ash A**	PFOA	
**Coal Fly-Ash A**	PFDA	
**Coal Fly-Ash A**	PFDoDA	
**Coal Fly-Ash B**	PFBA	
**Coal Fly-Ash B**	PFHxA	
**Coal Fly-Ash B**	PFOA	
**Coal Fly-Ash B**	PFDA	
**Coal Fly-Ash B**	PFDoDA	31
**Coal Fly-Ash C**	PFBA	
**Coal Fly-Ash C**	PFHxA	
**Coal Fly-Ash C**	PFOA	
**Coal Fly-Ash C**	PFDA	
**Coal Fly-Ash C**	PFDoDA	
**GAC Filtrasorb 400**	PFBA	
**GAC Filtrasorb 400**	PFHxA	
**GAC Filtrasorb 400**	PFOA	
**GAC Filtrasorb 400**	PFDA	
**GAC Filtrasorb 400**	PFDoDA	

**PAC**	PFBA
**PAC**	PFHxA
**PAC**	PFOA
**PAC**	PFDA
**PAC**	PFDoDA

Model Performance

Table S3: Model Performance for the 80/20 Validation

Model	R^2	RMSE (L/g)	MSE	MAE (L/g)
**Stand-Alone**				
**RSM**	0.29	0.77	0.60	0.57
**HOP-RSM**	0.58	0.60	0.35	0.44
**RF**	0.92	0.25	0.06	0.16
**GB**	0.93	0.25	0.06	0.15
**XGB**	0.91	0.27	0.07	0.17
**Linear Hybrid**				
**RSM-RF**	0.74	0.46	0.22	0.34
**RSM-GB**	0.78	0.43	0.19	0.32
**RSM-XGB**	0.74	0.47	0.22	0.34
**HOP-RF**	0.82	0.39	0.15	0.28
**HOP-GB**	0.85	0.36	0.13	0.25
**HOP-XGB**	0.81	0.39	0.16	0.28
**RMSE Hybrid**				
**RSM-RF**	0.27	0.78	0.61	0.58
**RSM-GB**	0.66	0.54	0.29	0.40
**RSM-XGB**	0.28	0.78	0.60	0.57
**HOP-RF**	0.31	0.76	0.58	0.56
**HOP-GB**	0.68	0.52	0.27	0.38
**HOP-XGB**	0.32	0.76	0.57	0.56
**Multiplicative Hybrid**				
**RSM-RF**	0.27	0.78	0.61	0.58
**RSM-GB**	0.66	0.54	0.29	0.40
**RSM-XGB**	0.28	0.78	0.60	0.57
**HOP-RF**	0.31	0.76	0.58	0.56
**HOP-GB**	0.68	0.52	0.27	0.38
**HOP-XGB**	0.32	0.76	0.57	0.56
**Meta Hybrid**				
**RSM-RF**	0.94	0.22	0.05	0.13
**RSM-GB**	0.93	0.25	0.06	0.14
**RSM-XGB**	0.92	0.26	0.07	0.14
**HOP-RF**	0.93	0.24	0.06	0.14

**HOP-GB**	0.93	0.25	0.06	0.14
**HOP-XGB**	0.92	0.26	0.07	0.15

Table S4: Model Performance for the LOPO Validation

Model	R^2	RMSE (L/g)	MSE	MAE (L/g)
**Stand-Alone**				
**RSM**	-23874879.08	4546.26	20668447.78	1172.09
**HOP-RSM**	-228537148.49	14065.71	197844266.61	8951.34
**RF**	0.30	0.78	0.61	0.57
**GB**	-0.36	1.08	1.18	0.81
**XGB**	0.10	0.88	0.78	0.65
**Linear Hybrid**				
**RSM-RF**	-5968469.49	2273.08	5166895.93	586.07
**RSM-GB**	-5968293.57	2273.05	5166743.63	586.09
**RSM-XGB**	-5968442.49	2273.08	5166872.56	586.05
**HOP-RF**	-57133969.87	7032.84	49460792.66	4475.68
**HOP-GB**	-57133019.29	7032.78	49459969.74	4475.62
**HOP-XGB**	-57134049.65	7032.84	49460861.73	4475.68
**RMSE Hybrid**				
**RSM-RF**	-70520.92	247.08	61050.72	59.65
**RSM-GB**	-70496.02	247.04	61029.17	59.65
**RSM-XGB**	-70518.37	247.08	61048.52	59.63
**HOP-RF**	-1426692.58	1111.34	1235086.49	707.61
**HOP-GB**	-1426365.73	1111.22	1234803.53	707.45
**HOP-XGB**	-1426676.19	1111.34	1235072.29	707.59
**Multiplicative Hybrid**				
**RSM-RF**	N/A	N/A	N/A	N/A
**RSM-GB**	N/A	N/A	N/A	N/A
**RSM-XGB**	N/A	N/A	N/A	N/A
**HOP-RF**	N/A	N/A	N/A	N/A
**HOP-GB**	N/A	N/A	N/A	N/A
**HOP-XGB**	N/A	N/A	N/A	N/A
**Meta Hybrid**				
**RSM-RF**	0.10	0.88	0.78	0.68
**RSM-GB**	-0.46	1.12	1.26	0.83
**RSM-XGB**	-0.15	1.00	1.00	0.75
**HOP-RF**	0.08	0.89	0.80	0.71
**HOP-GB**	-0.40	1.10	1.21	0.84
**HOP-XGB**	-0.33	1.07	1.15	0.77

Predicted vs Actual plots for all models

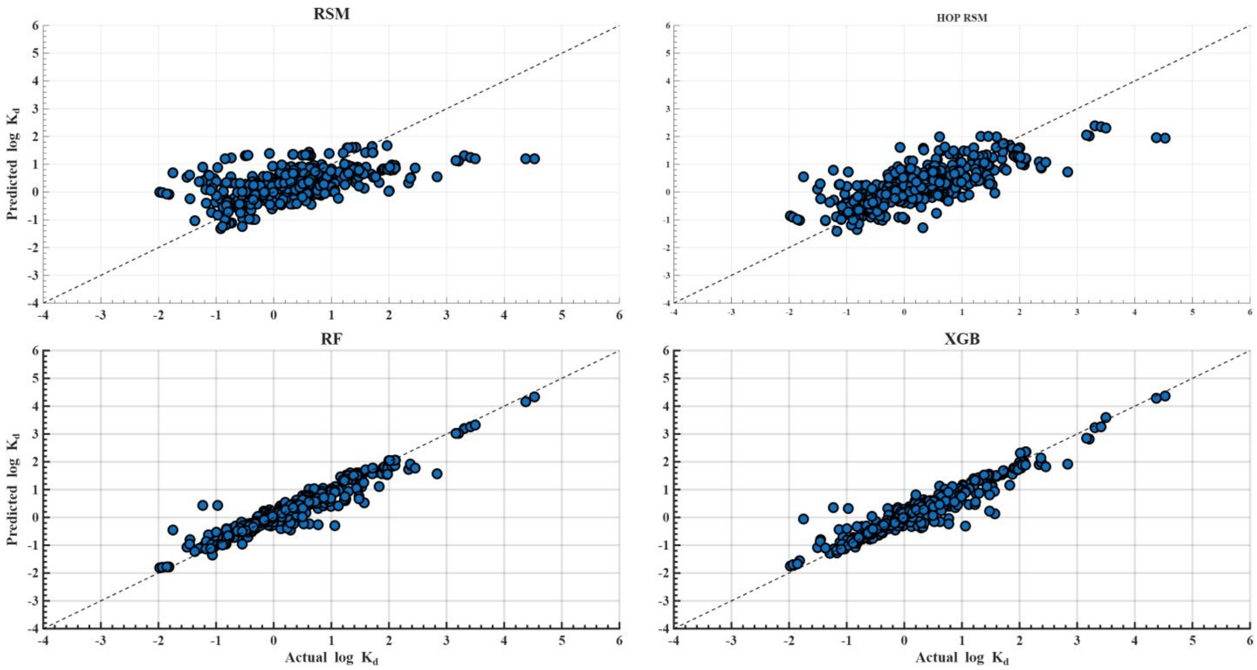

Figure S1: Predicted vs Actual Plot for Stand-Alone Models for 80/20 Validation

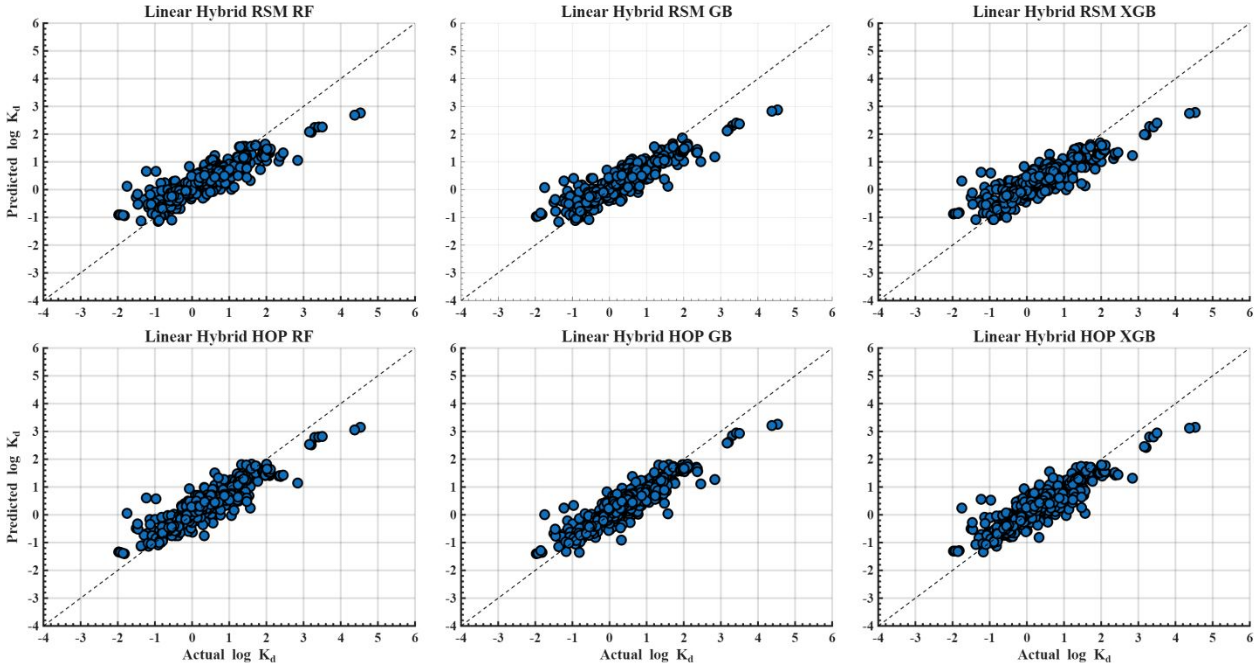

Figure S2: Predicted vs Actual Plot for Linear Hybrid Models for 80/20 Validation

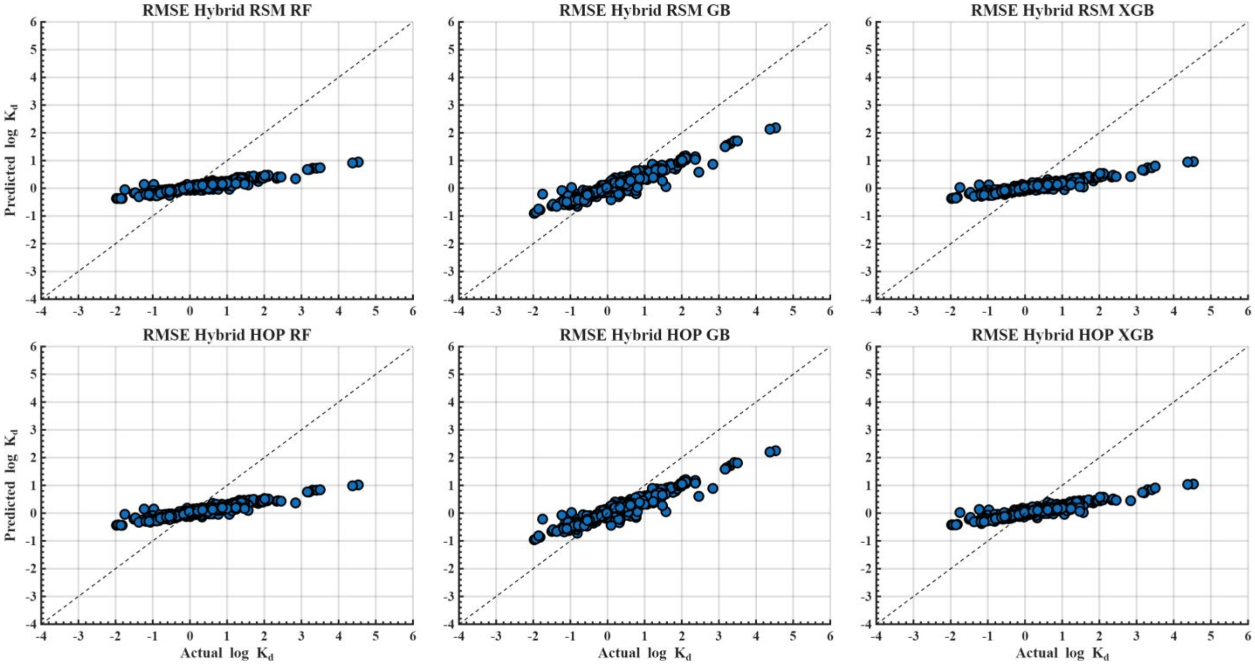

Figure S3: Predicted vs Actual Plot for RMSE Hybrid Models for 80/20 Validation

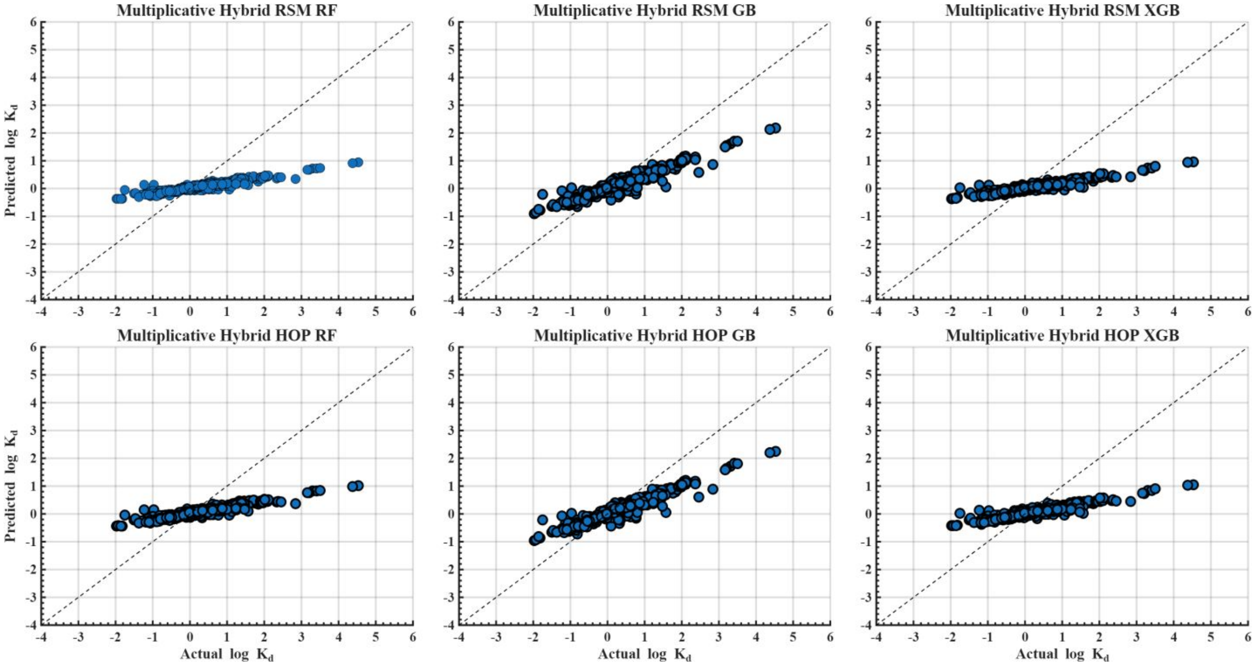

Figure S4: Predicted vs Actual Plot for Multiplicative Hybrid Models for 80/20 Validation

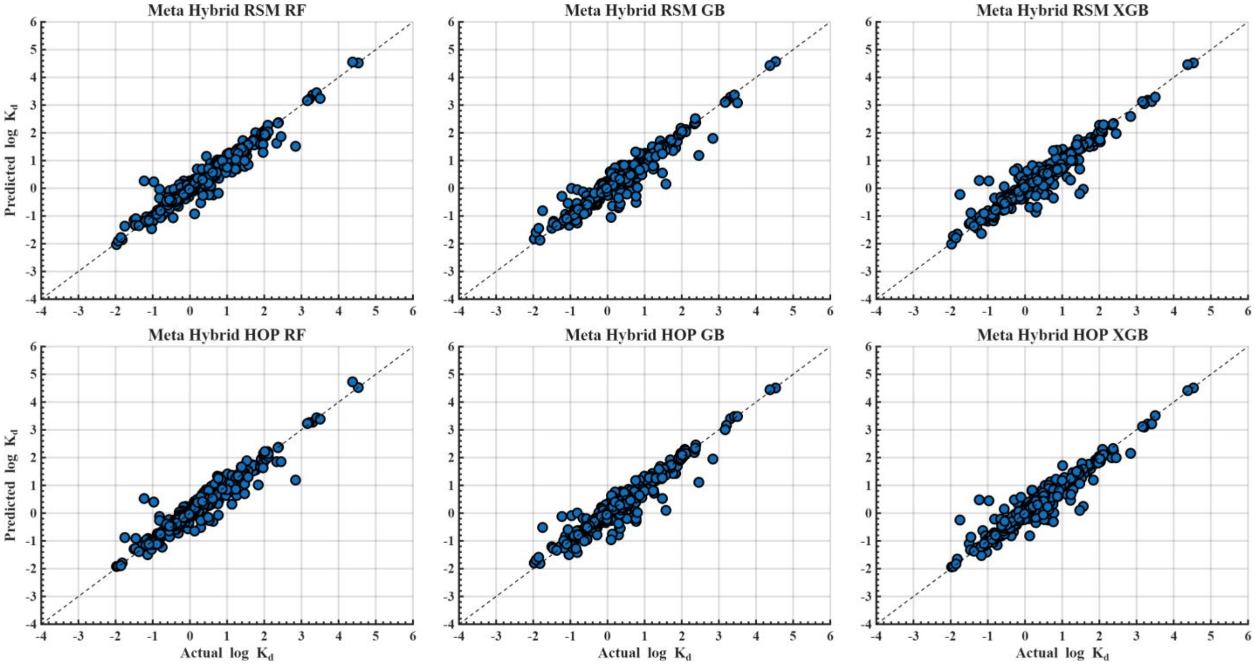

Figure S5: Predicted vs Actual Plot for Meta Hybrid Models for 80/20 Validation

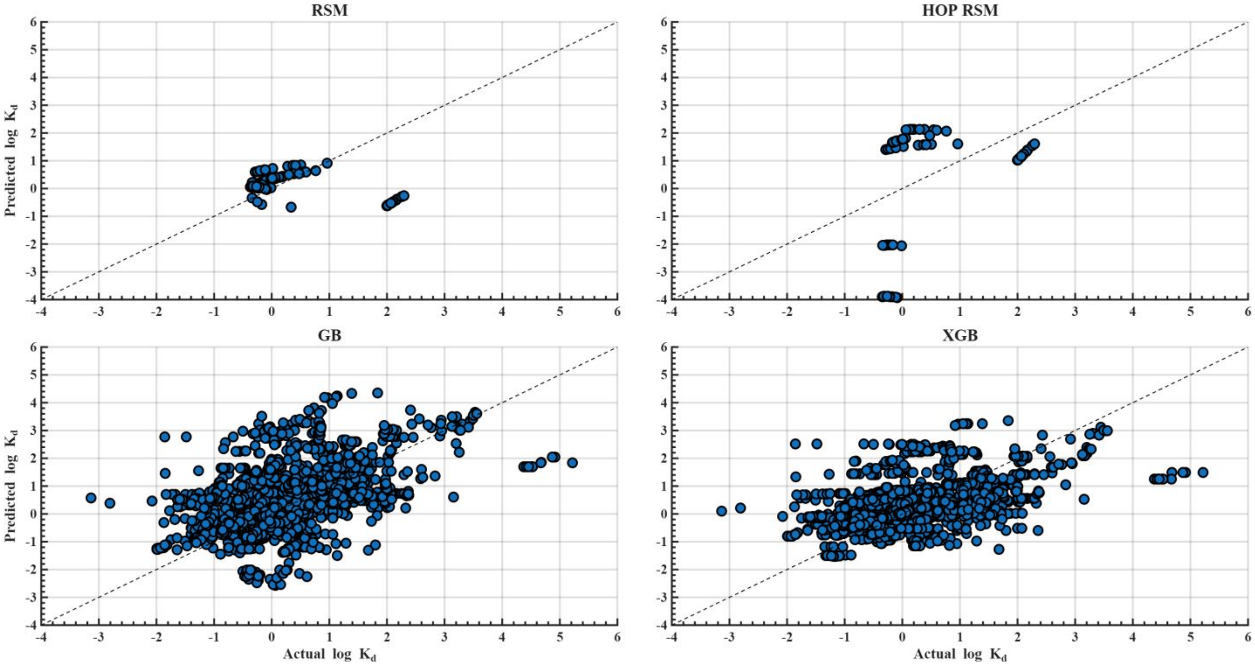

Figure S6: Predicted vs Actual Plot for Stand-Alone Models for LOPO Validation

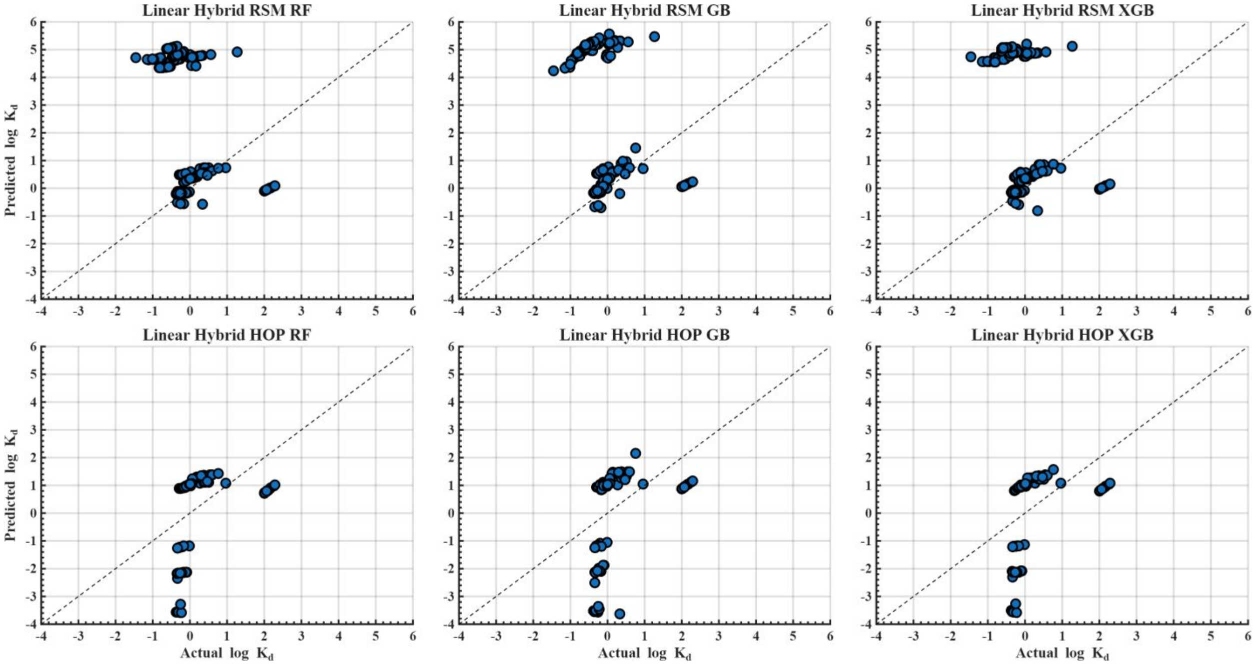

Figure S7: Predicted vs Actual Plot for Linear Hybrid Models for LOPO Validation

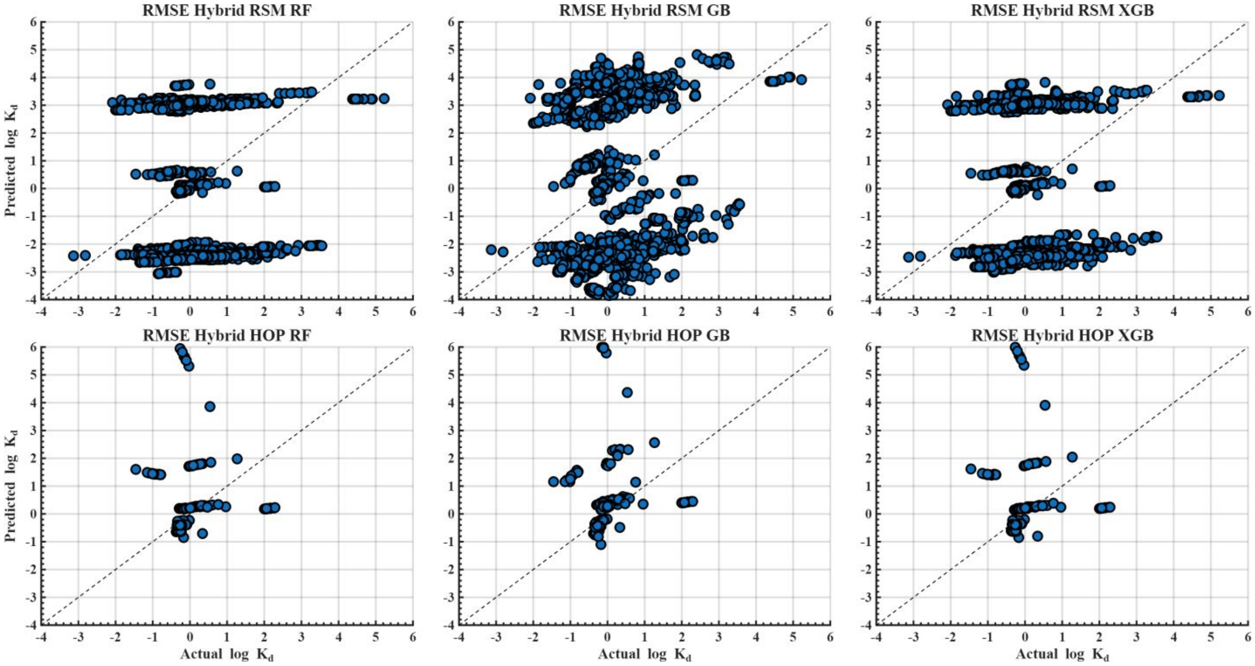

Figure S8: Predicted vs Actual Plot for RMSE Hybrid Models for LOPO Validation

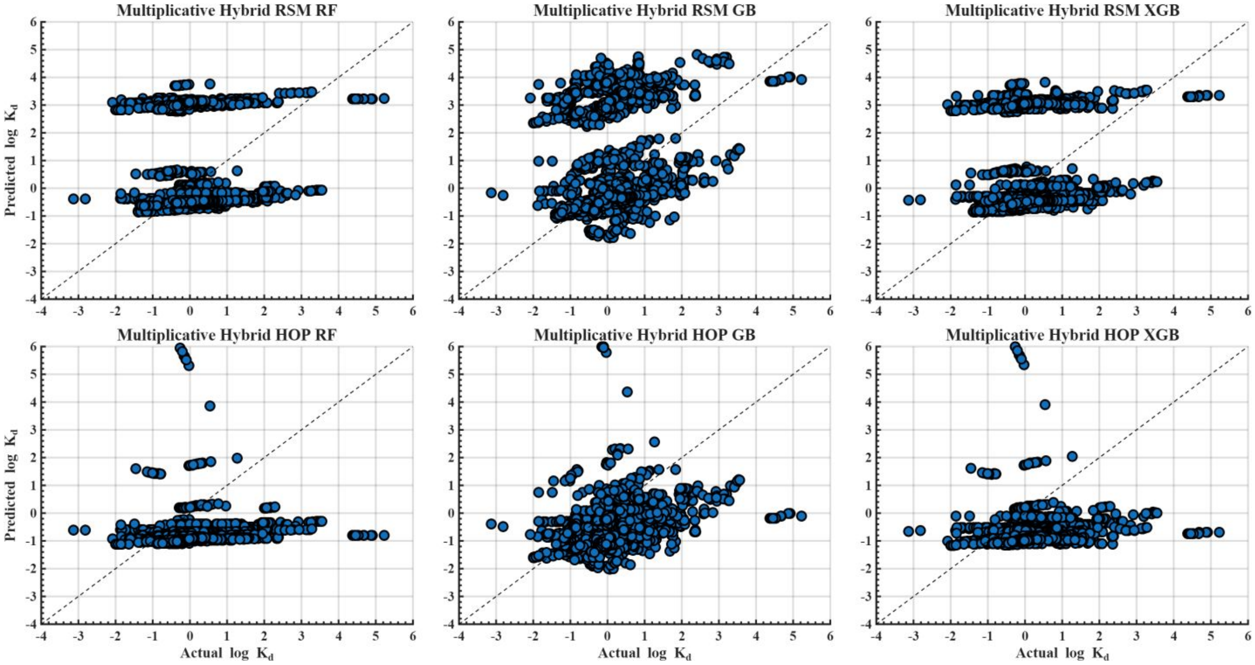

Figure S9: Predicted vs Actual Plot for Multiplicative Hybrid Models for LOPO Validation

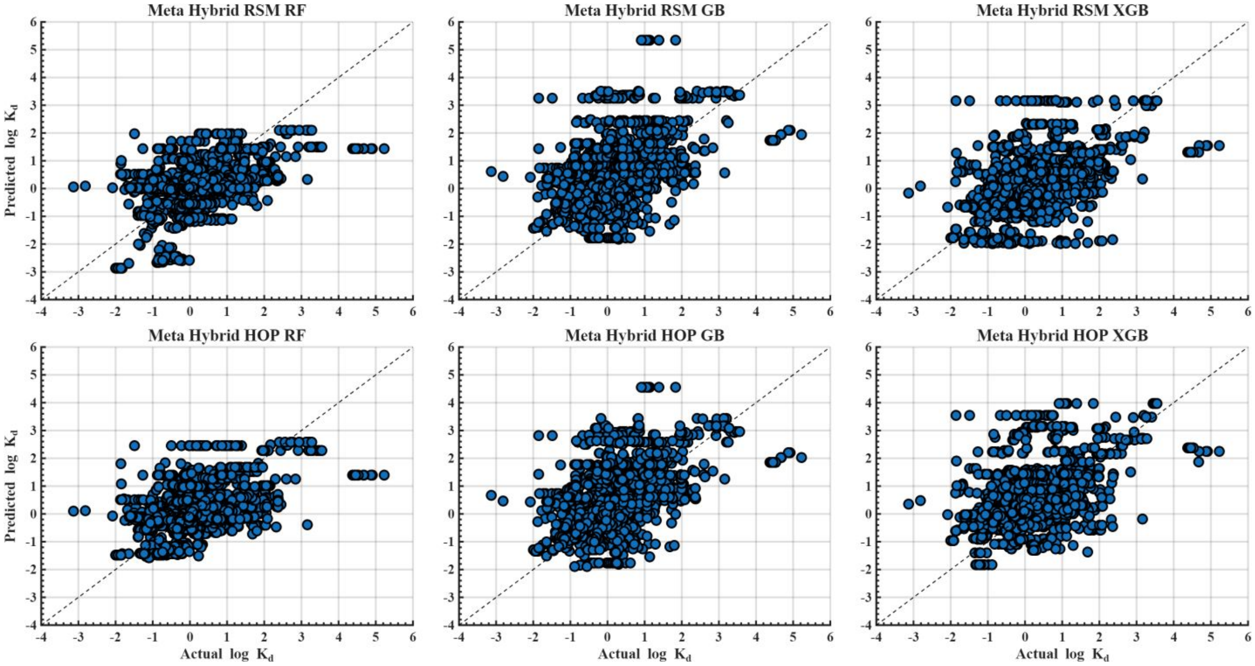

Figure S10: Predicted vs Actual Plot for Meta Hybrid Models for LOPO Validation

Residuals

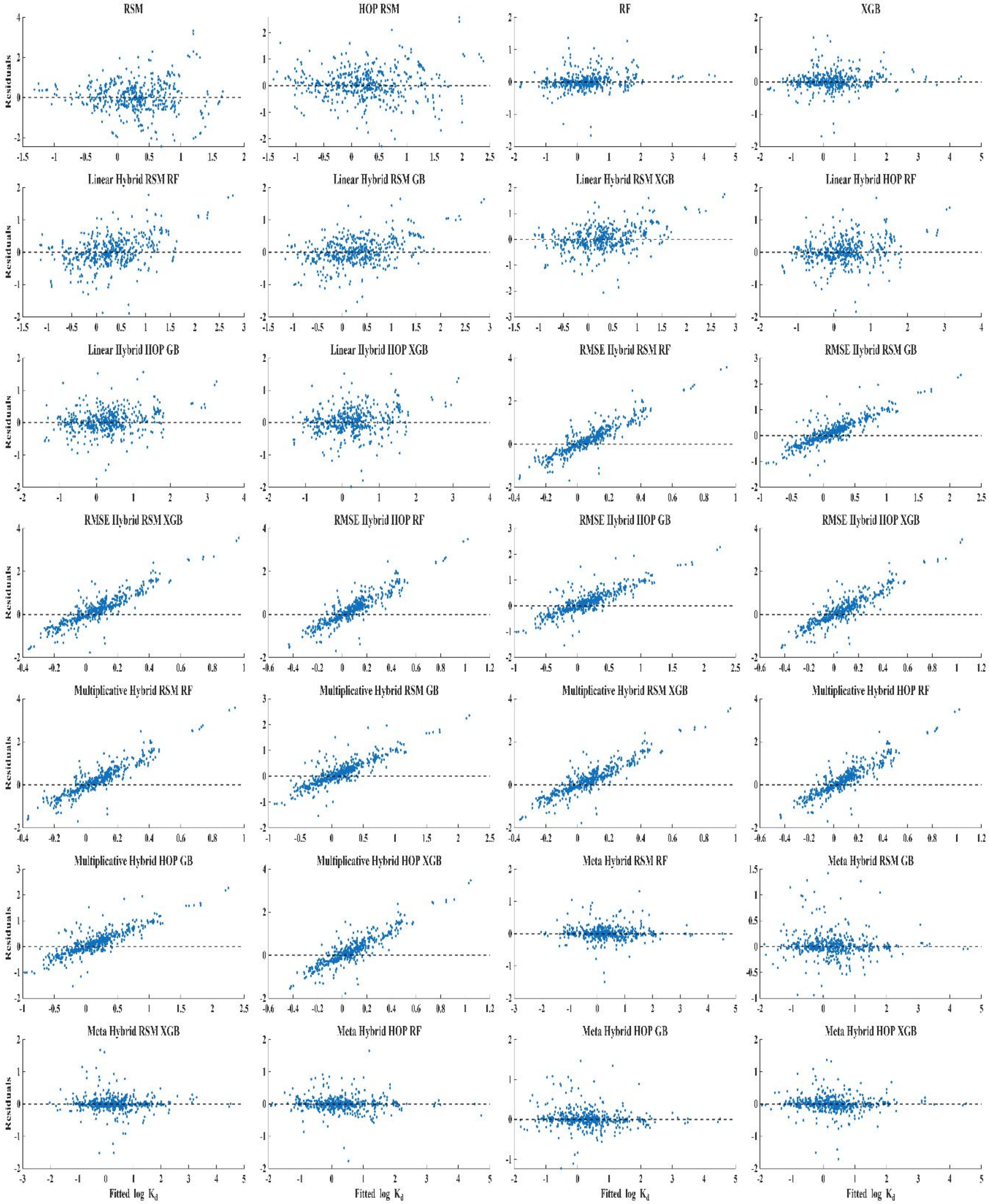

Figure S11: Residuals vs Fitted Log K_d Plot for all Models under 80/20 Validation

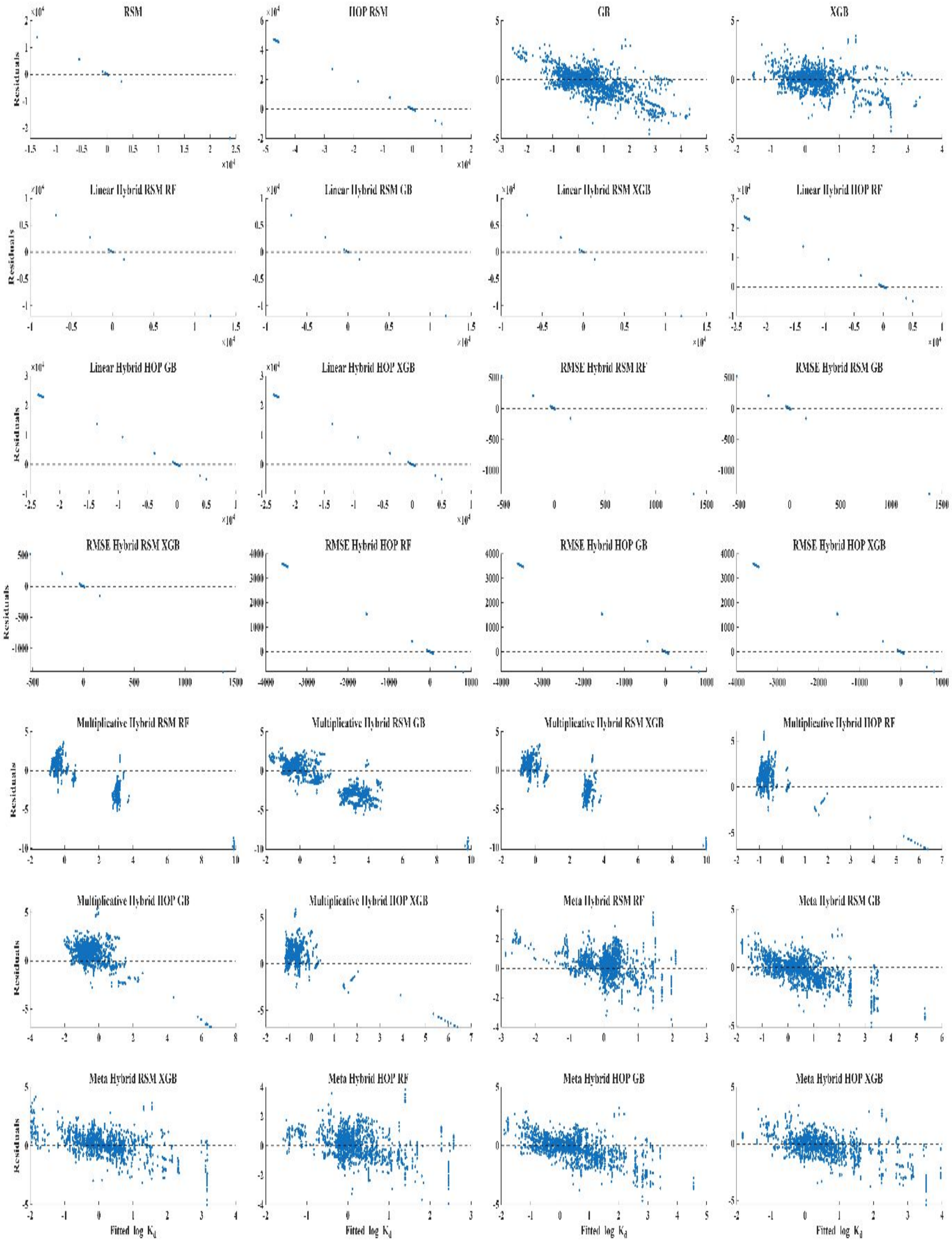

Figure S12: Residuals vs Fitted Log K_d Plot for all Models under LOPO Validation

Surface Plots

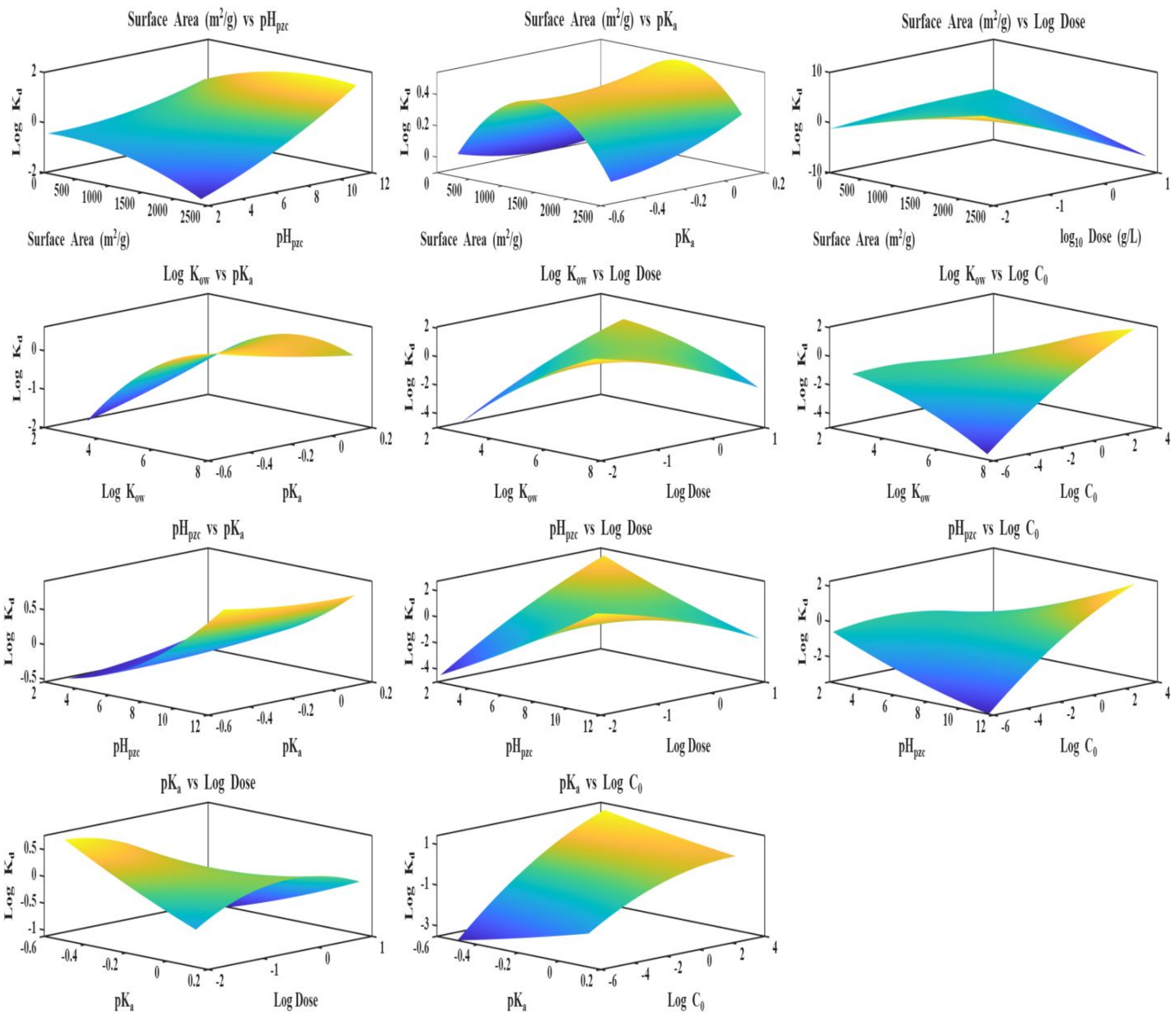

Figure S13: 3D Surface Plots for Log K_d with different Pairs of Descriptors.

Acknowledgements

The study was funded by National Science Foundation (CBET 2216148) and North Carolina Collaboratory Funds ID: Collab_493.

References

(1) Yu, Q.; Zhang, R.; Deng, S.; Huang, J.; Yu, G. Sorption of Perfluorooctane Sulfonate and Perfluorooctanoate on Activated Carbons and Resin: Kinetic and Isotherm Study. *Water Res.* **2009**, *43* (4), 1150–1158. https://doi.org/10.1016/j.watres.2008.12.001.

(2) Chen, X.; Xia, X.; Wang, X.; Qiao, J.; Chen, H. A Comparative Study on Sorption of Perfluorooctane Sulfonate (PFOS) by Chars, Ash and Carbon Nanotubes. *Chemosphere* **2011**, *83* (10), 1313–1319. https://doi.org/10.1016/j.chemosphere.2011.04.018.

(3) Chen, X.; Xia, X.; Wang, X.; Qiao, J.; Chen, H. A Comparative Study on Sorption of Perfluorooctane Sulfonate (PFOS) by Chars, Ash and Carbon Nanotubes. *Chemosphere* **2011**, *83* (10), 1313–1319. https://doi.org/10.1016/j.chemosphere.2011.04.018.

(4) Lei, X.; Lian, Q.; Zhang, X.; Wang, T.; Gee, M.; Holmes, W.; Jin, S.; Ponnusamy, S. K.; Gang, D. D.; Zappi, M. E. Removal of Perfluorooctanoic Acid via Polyethyleneimine Modified Graphene Oxide: Effects of Water Matrices and Understanding Mechanisms. *Chemosphere* **2022**, *308*, 136379. https://doi.org/10.1016/j.chemosphere.2022.136379.

(5) Ateia, M.; Attia, M. F.; Maroli, A.; Tharayil, N.; Alexis, F.; Whitehead, D. C.; Karanfil, T. Rapid Removal of Poly- and Perfluorinated Alkyl Substances by Poly(Ethylenimine)-Functionalized Cellulose Microcrystals at Environmentally Relevant Conditions. *Environ. Sci. Technol. Lett.* **2018**, *5* (12), 764–769. https://doi.org/10.1021/acs.estlett.8b00556.

(6) Lei, X.; Yao, L.; Lian, Q.; Zhang, X.; Wang, T.; Holmes, W.; Ding, G.; Gang, D. D.; Zappi, M. E. Enhanced Adsorption of Perfluorooctanoate (PFOA) onto Low Oxygen Content Ordered Mesoporous Carbon (OMC): Adsorption Behaviors and Mechanisms. *J. Hazard. Mater.* **2022**, *421*, 126810. https://doi.org/10.1016/j.jhazmat.2021.126810.

(7) Deng, S.; Zhang, Q.; Nie, Y.; Wei, H.; Wang, B.; Huang, J.; Yu, G.; Xing, B. Sorption Mechanisms of Perfluorinated Compounds on Carbon Nanotubes. *Environ. Pollut.* **2012**, *168*, 138–144. https://doi.org/10.1016/j.envpol.2012.03.048.

(8) Liu, L.; Li, D.; Li, C.; Ji, R.; Tian, X. Metal Nanoparticles by Doping Carbon Nanotubes Improved the Sorption of Perfluorooctanoic Acid. *J. Hazard. Mater.* **2018**, *351*, 206–214. https://doi.org/10.1016/j.jhazmat.2018.03.001.

(9) Zhou, Q.; Deng, S.; Yu, Q.; Zhang, Q.; Yu, G.; Huang, J.; He, H. Sorption of Perfluorooctane Sulfonate on Organo-Montmorillonites. *Chemosphere* **2010**, *78* (6), 688–694. https://doi.org/10.1016/j.chemosphere.2009.12.005.

(10) Xing, D. Y.; Chen, Y.; Zhu, J.; Liu, T. Fabrication of Hydrolytically Stable Magnetic Core-Shell Aminosilane Nanocomposite for the Adsorption of PFOS and PFOA. *Chemosphere* **2020**, *251*, 126384. https://doi.org/10.1016/j.chemosphere.2020.126384.

(11) Liu, T.; Gu, Y.; Xing, D. Y.; Dong, W.; Wu, X. Rapid and High-Capacity Adsorption of PFOS and PFOA by Regenerable Ammoniated Magnetic Particle. *Environ. Sci. Pollut. Res.* **2018**, *25* (14), 13813–13822. https://doi.org/10.1007/s11356-018-1578-1.

(12) Chang, P.-H.; Jiang, W.-T.; Li, Z. Removal of Perfluorooctanoic Acid from Water Using Calcined Hydrotalcite – A Mechanistic Study. *J. Hazard. Mater.* **2019**, *368*, 487–495. https://doi.org/10.1016/j.jhazmat.2019.01.084.

(13) Chang, P.-H.; Li, Z.; Jiang, W.-T. Calcination of Hydrotalcite to Enhance the Removal of Perfluorooctane Sulfonate from Water. *Appl. Clay Sci.* **2020**, *190*, 105563. https://doi.org/10.1016/j.clay.2020.105563.

(14) Zhang, Q.; Deng, S.; Yu, G.; Huang, J. Removal of Perfluorooctane Sulfonate from Aqueous Solution by Crosslinked Chitosan Beads: Sorption Kinetics and Uptake Mechanism. *Bioresour. Technol.* **2011**, *102* (3), 2265–2271. https://doi.org/10.1016/j.biortech.2010.10.040.

(15) Yang, A.; Ching, C.; Easler, M.; Helbling, D. E.; Dichtel, W. R. Cyclodextrin Polymers with Nitrogen-Containing Tripodal Crosslinkers for Efficient PFAS Adsorption. *ACS Mater. Lett.* **2020**, *2* (9), 1240–1245. https://doi.org/10.1021/acsmaterialslett.0c00240.

(16) Senevirathna, S. T. M. L. D.; Tanaka, S.; Fujii, S.; Kunacheva, C.; Harada, H.; Shivakoti, B. R.; Okamoto, R. A Comparative Study of Adsorption of Perfluorooctane Sulfonate (PFOS) onto Granular Activated Carbon, Ion-Exchange Polymers and Non-Ion-Exchange Polymers. *Chemosphere* **2010**, *80* (6), 647–651. https://doi.org/10.1016/j.chemosphere.2010.04.053.
(17) Chen, W.; Zhang, X.; Mamadiev, M.; Wang, Z. Sorption of Perfluorooctane Sulfonate and Perfluorooctanoate on Polyacrylonitrile Fiber-Derived Activated Carbon Fibers: In Comparison with Activated Carbon. *RSC Adv.* **2017**, *7* (2), 927–938. https://doi.org/10.1039/C6RA25230C.
(18) Fagbayigbo, B. O.; Opeolu, B. O.; Fatoki, O. S.; Akenga, T. A.; Olatunji, O. S. Removal of PFOA and PFOS from Aqueous Solutions Using Activated Carbon Produced from Vitis Vinifera Leaf Litter. *Environ. Sci. Pollut. Res.* **2017**, *24* (14), 13107–13120. https://doi.org/10.1007/s11356-017-8912-x.
(19) Zhang, D.; Luo, Q.; Gao, B.; Chiang, S.-Y. D.; Woodward, D.; Huang, Q. Sorption of Perfluorooctanoic Acid, Perfluorooctane Sulfonate and Perfluoroheptanoic Acid on Granular Activated Carbon. *Chemosphere* **2016**, *144*, 2336–2342. https://doi.org/10.1016/j.chemosphere.2015.10.124.
(20) Meng, P.; Fang, X.; Maimaiti, A.; Yu, G.; Deng, S. Efficient Removal of Perfluorinated Compounds from Water Using a Regenerable Magnetic Activated Carbon. *Chemosphere* **2019**, *224*, 187–194. https://doi.org/10.1016/j.chemosphere.2019.02.132.
(21) Qian, J.; Shen, M.; Wang, P.; Wang, C.; Li, K.; Liu, J.; Lu, B.; Tian, X. Perfluorooctane Sulfonate Adsorption on Powder Activated Carbon: Effect of Phosphate (P) Competition, pH, and Temperature. *Chemosphere* **2017**, *182*, 215–222. https://doi.org/10.1016/j.chemosphere.2017.05.033.
(22) Zhi, Y.; Liu, J. Surface Modification of Activated Carbon for Enhanced Adsorption of Perfluoroalkyl Acids from Aqueous Solutions. *Chemosphere* **2016**, *144*, 1224–1232. https://doi.org/10.1016/j.chemosphere.2015.09.097.
(23) Wang, B.; Lee, L. S.; Wei, C.; Fu, H.; Zheng, S.; Xu, Z.; Zhu, D. Covalent Triazine-Based Framework: A Promising Adsorbent for Removal of Perfluoroalkyl Acids from Aqueous Solution. *Environ. Pollut.* **2016**, *216*, 884–892. https://doi.org/10.1016/j.envpol.2016.06.062.
(24) Wang, F.; Liu, C.; Shih, K. Adsorption Behavior of Perfluorooctanesulfonate (PFOS) and Perfluorooctanoate (PFOA) on Boehmite. *Chemosphere* **2012**, *89* (8), 1009–1014. https://doi.org/10.1016/j.chemosphere.2012.06.071.
(25) Wang, F.; Shih, K. Adsorption of Perfluorooctanesulfonate (PFOS) and Perfluorooctanoate (PFOA) on Alumina: Influence of Solution pH and Cations. *Water Res.* **2011**, *45* (9), 2925–2930. https://doi.org/10.1016/j.watres.2011.03.007.
(26) Jian, J.-M.; Zhang, C.; Wang, F.; Lu, X.; Wang, F.; Zeng, E. Y. Effect of Solution Chemistry and Aggregation on Adsorption of Perfluorooctanesulphonate (PFOS) to Nano-Sized Alumina. *Environ. Pollut.* **2019**, *251*, 425–433. https://doi.org/10.1016/j.envpol.2019.05.025.
(27) Tang, C. Y.; Shiang Fu, Q.; Gao, D.; Criddle, C. S.; Leckie, J. O. Effect of Solution Chemistry on the Adsorption of Perfluorooctane Sulfonate onto Mineral Surfaces. *Water Res.* **2010**, *44* (8), 2654–2662. https://doi.org/10.1016/j.watres.2010.01.038.
(28) Yoon, S.; Park, J.; Police, A. K. R.; Choe, J. K.; Bae, S. Enhanced Removal of Perfluorooctanoic Acid by Aluminum-Based Metal–Organic Frameworks Prepared by

Bauxite Residue. *J. Hazard. Mater.* **2025**, *483*, 136687. https://doi.org/10.1016/j.jhazmat.2024.136687.

(29) Zhao, L.; Bian, J.; Zhang, Y.; Zhu, L.; Liu, Z. Comparison of the Sorption Behaviors and Mechanisms of Perfluorosulfonates and Perfluorocarboxylic Acids on Three Kinds of Clay Minerals. *Chemosphere* **2014**, *114*, 51–58. https://doi.org/10.1016/j.chemosphere.2014.03.098.

(30) Campos-Pereira, H.; Kleja, D. B.; Sjöstedt, C.; Ahrens, L.; Klysubun, W.; Gustafsson, J. P. The Adsorption of Per- and Polyfluoroalkyl Substances (PFASs) onto Ferrihydrite Is Governed by Surface Charge. *Environ. Sci. Technol.* **2020**, *54* (24), 15722–15730. https://doi.org/10.1021/acs.est.0c01646.

(31) Patel, H. V.; Greer, M.; Brazil, B.; Yu, W.; Hamoush, S.; Zhang, L.; Zhao, R. Adsorption Capacity and Mechanism of Modified Coal Fly Ash (CFA) for per- and Polyfluoroalkyl Substances (PFAS) in Landfill Leachate. *J. Hazard. Mater.* **2025**, *484*, 136763. https://doi.org/10.1016/j.jhazmat.2024.136763.
